# Spatiotemporal DNA methylome dynamics of the developing mouse fetus

**DOI:** 10.1038/s41586-020-2119-x

**Published:** 2020-07-29

**Authors:** Yupeng He, Manoj Hariharan, David U. Gorkin, Diane E. Dickel, Chongyuan Luo, Rosa G. Castanon, Joseph R. Nery, Ah Young Lee, Yuan Zhao, Hui Huang, Brian A. Williams, Diane Trout, Henry Amrhein, Rongxin Fang, Huaming Chen, Bin Li, Axel Visel, Len A. Pennacchio, Bing Ren, Joseph R. Ecker

**Affiliations:** 1 Genomic Analysis Laboratory, The Salk Institute for Biological Studies, La Jolla, CA USA; 2 Bioinformatics and Systems Biology Program, University of California, San Diego, La Jolla, CA USA; 3 Ludwig Institute for Cancer Research, University of California, San Diego, La Jolla, CA USA; 4 Environmental Genomics and Systems Biology Division, Lawrence Berkeley National Laboratory, Berkeley, CA USA; 5 Biomedical Sciences Graduate Program, University of California, San Diego, La Jolla, CA USA; 6 Division of Biology and Biological Engineering, California Institute of Technology, Pasadena, CA USA; 7 US Department of Energy Joint Genome Institute, Lawrence Berkeley National Laboratory, Berkeley, CA USA; 8 School of Natural Sciences, University of California, Merced, Merced, CA USA; 9 Comparative Biochemistry Program, University of California, Berkeley, CA USA; 10 Department of Cellular and Molecular Medicine, University of California, San Diego, La Jolla, CA USA; 11 Howard Hughes Medical Institute, The Salk Institute for Biological Studies, La Jolla, CA USA

**Keywords:** Epigenomics, DNA methylation, Gene regulation

## Abstract

Cytosine DNA methylation is essential for mammalian development but understanding of its spatiotemporal distribution in the developing embryo remains limited^1,2^. Here, as part of the mouse Encyclopedia of DNA Elements (ENCODE) project, we profiled 168 methylomes from 12 mouse tissues or organs at 9 developmental stages from embryogenesis to adulthood. We identified 1,808,810 genomic regions that showed variations in CG methylation by comparing the methylomes of different tissues or organs from different developmental stages. These DNA elements predominantly lose CG methylation during fetal development, whereas the trend is reversed after birth. During late stages of fetal development, non-CG methylation accumulated within the bodies of key developmental transcription factor genes, coinciding with their transcriptional repression. Integration of genome-wide DNA methylation, histone modification and chromatin accessibility data enabled us to predict 461,141 putative developmental tissue-specific enhancers, the human orthologues of which were enriched for disease-associated genetic variants. These spatiotemporal epigenome maps provide a resource for studies of gene regulation during tissue or organ progression, and a starting point for investigating regulatory elements that are involved in human developmental disorders.

## Abstract (web summary)

Analysis of 168 methylomes from 12 mouse tissues at 9 developmental stages sheds light on the epigenetic and regulatory landscape during mammalian fetal development.

## Main

Mammalian embryonic development involves exquisite spatiotemporal regulation of genes^1,3,4^. This process is mediated by the sophisticated orchestration of transcription factors (TFs) that bind to regulatory DNA elements (primarily enhancers and promoters) and epigenetic modifications that influence these events. Specifically, the ability of TFs to access regulatory DNA is closely related to the covalent modification of histones and DNA^5,6^.

Cytosine DNA methylation is an epigenetic modification that is crucial for gene regulation^2^. This base modification occurs predominantly at cytosines followed by guanine (mCG) in mammalian genomes and is dynamic at regulatory elements in different tissues and cell types^7–11^. mCG can directly affect the DNA-binding affinity of a variety of TFs^6,12^ and targeted addition or removal of mCG at promoters correlates with increases or decreases, respectively, in gene transcription^13^. Non-CG methylation (mCH; in which H denotes A, C or T) is also present at appreciable levels in embryonic stem cells, oocytes, heart and skeletal muscle, and is abundant in the mammalian brain^7–9,11,14–17^. In fact, the level of mCH in human neurons exceeds that of mCG^9^. Although its precise function(s) are unknown, mCH directly affects DNA binding by MeCP2, the methyl-binding protein in which mutations are responsible for Rett syndrome^18^.

Cytosine DNA methylation is actively regulated during mammalian development^19^. However, compared to pre-implantation embryogenesis^19–21^, epigenomic data are lacking for later stages, during which anatomical features of the major organ systems emerge and human birth defects become manifest^22^. To fill this knowledge gap, as part of the mouse ENCODE project, we used the mouse embryo to generate epigenomic and transcriptomic maps for twelve tissue types at nine developmental stages from embryonic day 10.5 (E10.5) to birth (postnatal day 0, P0) and, for some tissues, to adulthood. We performed whole-genome bisulfite sequencing (WGBS) to generate base-resolution methylome maps. In other papers published as part of ENCODE^23,24^, the same tissue samples were profiled using chromatin immunoprecipitation with sequencing (ChIP–seq), assay for transposase-accessible chromatin data using sequencing (ATAC–seq)^23,25^ and RNA sequencing (RNA-seq)^24^ to identify histone modification, chromatin accessibility and gene expression landscapes, respectively.

These data sets allow the dynamics of gene regulation in developing fetal tissues to be studied, expanding the scope of the previous phase of mouse ENCODE^26^, which focused on gene regulation in adult tissues. These comprehensive data sets are publicly accessible at http://encodeproject.org and http://neomorph.salk.edu/ENCODE_mouse_fetal_development.html. Highlights of this paper include:

- Identification of 1,808,810 genomic regions showing developmental and tissue-specific mCG variation in fetal tissues, covering 22.5% of the mouse genome.
- Most (91.5%) of the mCG variant regions have no overlap with promoters, CpG islands or CpG island shores.
- The dominant methylation patterns observed were a continuous loss of CG demethylation prenatally during fetal progression, and CG remethylation postnatally, primarily at distal regulatory elements.
- During fetal development, non-CG methylation accumulated at the bodies of genes that encode developmental TFs, and this was associated with the future repression of these genes.
- We used integrative analyses of DNA methylation, histone modifications and chromatin accessibility data from mouse ENCODE to predict 461,141 putative enhancers across all fetal tissues.
- The putative fetal enhancers accurately recapitulate experimentally validated enhancers in matched tissue types from matched developmental stages.
- Predicted regulatory elements showed spatiotemporal enhancer-like active chromatin, which correlates with the dynamic expression patterns of genes that are essential for tissue development.
- The human orthologues of the fetal putative enhancers are enriched for genetic variants that are risk factors for a variety of human diseases.

## Developing fetal tissue methylomes

**Fig. 1:**
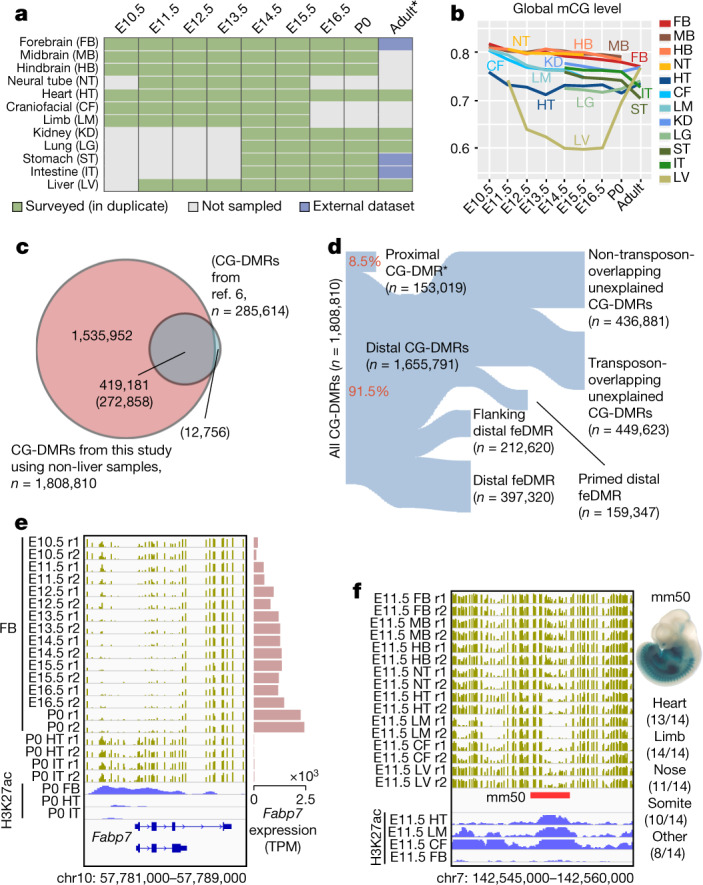
Annotation of methylation variable regulatory elements in developing mouse tissues. **a**, Tissue samples (green) profiled in this study. Blue cells indicate published data, grey cells indicate tissues and stages that were not sampled because either the organ is not yet formed or it was not possible to obtain sufficient material for the experiment, or the tissue was too heterogeneous to obtain informative data. *Additional data were generated in duplicate for adult tissues. **b**, Global mCG level of each tissue across their developmental trajectories. The adult forebrain was approximated using postnatal six-week-old frontal cortex^9^. **c**, Fetal CG-DMRs identified in this study encompass the majority of the adult CG-DMRs from a previous study^11^. Numbers with and without parentheses are related to fetal CG-DMRs and adult CG-DMRs, respectively. **d**, Categorization of CG-DMRs. Proximal CG-DMRs are those that overlap with promoters, CGIs or CGI shores. The rest are distal CG-DMRs. Fetal enhancer-linked CG-DMRs (feDMRs) are those that are predicted to be putative enhancers; those within 1 kb of distal feDMRs are flanking distal feDMRs. The remaining distal CG-DMRs showing hypomethylation are primed distal feDMRs. The rest are unexplained distal CG-DMRs, the functions of which are unknown, and they are further stratified according to their overlap with transposons (Methods). *Proximal CG-DMRs include 70,821 proximal feDMRs. **e**, mCG, H3K27ac and expression dynamics of *Fabp7*. Gold ticks represent CG sites; height represents mCG level, ranging from 0 to 1. The bottom three tracks show input-normalized H3K27ac enrichment in reads per kilobase per million mapped reads (RPKM), ranging from 0 to 20. *Fabp7* expression in transcripts per million mapped reads (TPM) is shown on the right. **f**, mCG and H3K27ac profiles near an experimentally validated enhancer from the VISTA enhancer data set^28^. The data on the right show the number of embryos in which the enhancer element (ID in VISTA: mm50) was active in a given tissue (out of a total *n* = 14 embryos). The image shows the tissue where the tested enhancer was active in one representative embryo. r1 and r2 denote first and second replicate, respectively.

To assess the cytosine DNA methylation landscape in the developing mouse embryo, we generated 168 methylomes to cover most of the major organ systems and tissue types derived from the 3 primordial germ layers (Fig. 1a). All methylomes exceeded ENCODE standards, with deep sequencing depth (median 31.8×) with biological replication, high conversion rate (over 99.5%) and high reproducibility; the Pearson correlation coefficient of mCG quantification between biological replicates is more than 0.8 (Supplementary Table 1, Methods). The reproducibility of liver methylomes is slightly lower because liver shows genome-wide hypomethylation, which causes higher sampling variation (Pearson correlation coefficient >0.73). To better understand the epigenomic landscape during fetal development, we also incorporated into our analyses histone modification (ChIP–seq), chromatin accessibility (ATAC–seq)^23^ and gene expression (RNA-seq) data^24^ from the same tissue and organ samples (Supplementary Table 2).

**Extended Data Fig. 1:**
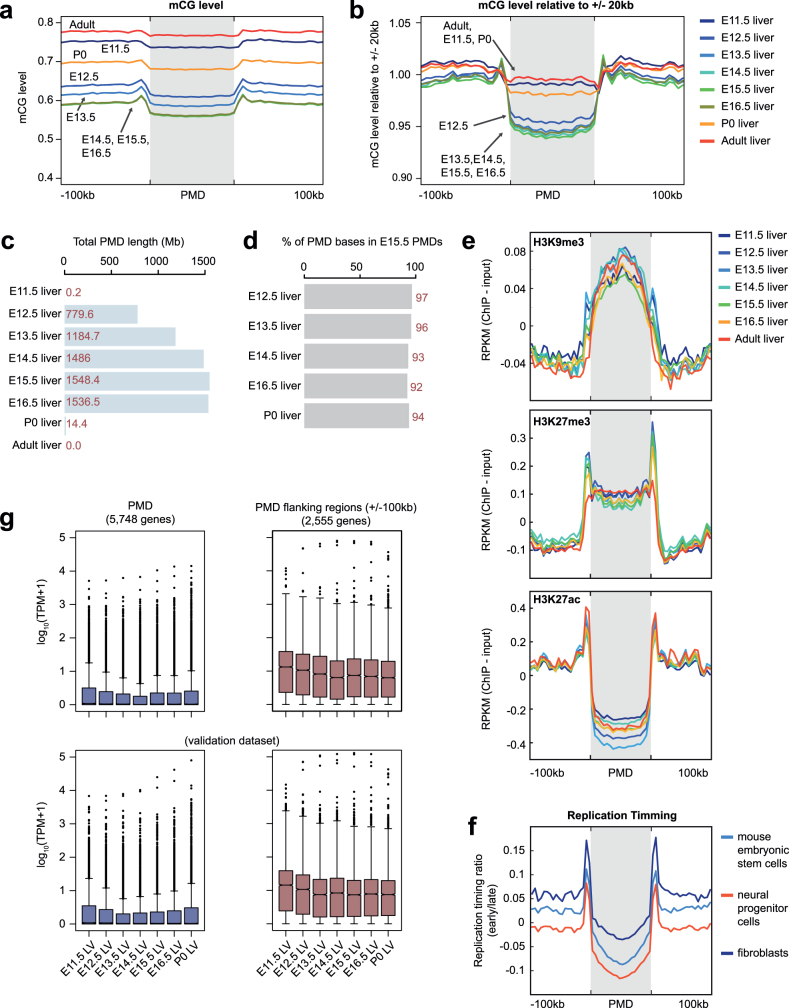
Global hypomethylation in fetal liver. **a**, Average mCG level of PMDs and flanking regions (±100 kb) in liver samples from different developmental stages. **b**, Normalized average mCG level of PMDs and flanking regions in liver samples. The mCG level was normalized (scaled) such that the average mCG level of ±20-kb regions around each PMD is 1.0. **c**, The total bases that PMDs encompass in liver at different developmental stages. **d**, Percentage of bases in PMDs identified in each of the liver samples (E12.5 liver, E13.5 liver and so on) that are also within PMDs identified in E15.5 liver sample. **e**, Histone modification profiles for H3K9me3 (top), H3K27me3 (middle) and H3K27ac (bottom) within PMDs and flanking regions (±100 kb) in liver samples from different developmental stages. **f**, Replication timing profiling of PMDs and flanking regions (±100 kb). The values indicate the tendency to be replicated at an earlier stage in the cell cycle. **g**, Expression of genes that overlap PMDs and flanking regions (±100 kb) (left) compared with those with no PMD overlap (right). Two plots below show data from a validation data set, containing RNA-seq data generated using a different protocol on matched tissues. Middle line, median; box, upper and lower quartiles; whiskers, 1.5 × (Q3 − Q1) above Q3 and below Q1; points, outliers.

The genomes of all fetal tissues were heavily CG methylated, with global mCG levels of 70–82% (with the notable exception of liver, 60–74%; Fig. 1b). Mouse fetal liver showed a signature of partially methylated domains (PMDs)^7^. Notably, the formation and dissolution of PMDs precisely coincided with fetal liver haematopoiesis (Supplementary Note 1, Extended Data Fig. 1).

**Extended Data Fig. 2:**
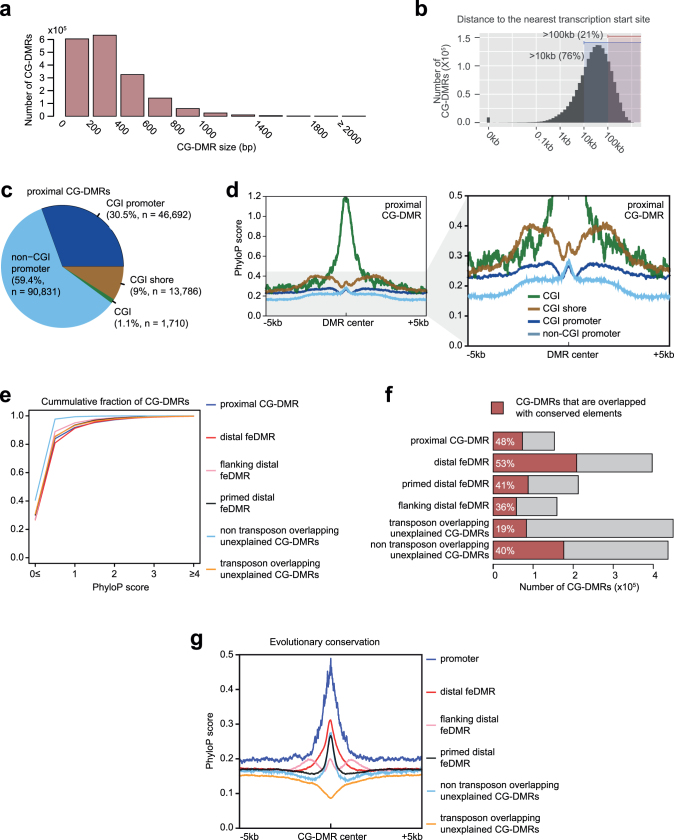
Categorization of CG-DMRs. **a**, CG-DMR size distribution. **b**, Distance from CG-DMRs to the nearest TSSs. **c**, Genomic distribution of proximal CG-DMRs. **d**, Evolutionary conservation of proximal CG-DMRs that overlap with CGIs, CGI shores, CGI promoters and non-CGI promoters. PhyloP score was used to measure the degree of conservation. **e**, Cumulative distribution of conservation score of CG-DMRs in different categories. **f**, Fraction of CG-DMRs in different categories that overlap with PhastCons conserved elements (Methods). **g**, Conservation (PhyloP) scores of promoters and different categories of distal CG-DMRs and flanking regions (±5 kb).

Although levels of global mCG were similar in fetal tissues at different stages, we identified 1,808,810 CG differentially methylated regions (CG-DMRs; genomic regions in which methylation differs between tissue types and developmental stages), which are, on average, 339 bp long and cover 22.5% (614 Mb) of the mouse genome (Extended Data Fig. 2a, Methods). This comprehensive fetal tissue CG-DMR annotation captured around 96% (*n* = 272,858) of all previously reported adult mouse tissue CG-DMRs^11^, and identified more than 1.5 million new regions (Fig. 1c).

**Extended Data Fig. 3:**
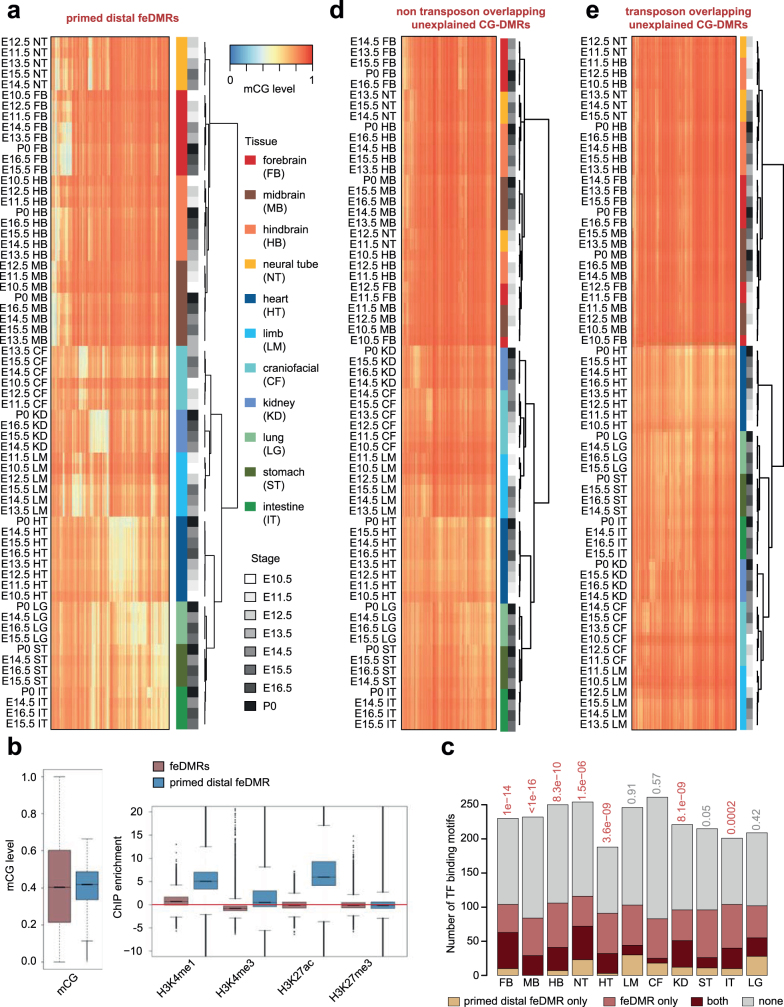
Characterization of primed distal feDMRs and unxDMRs. **a**, mCG level of all primed distal feDMRs in all non-liver tissues. Each row in the heatmap is one tissue sample and each column corresponds to one primed distal feDMR. Both rows and columns were clustered using hierarchical clustering. Coloured bars indicate tissue types and grey bars indicate developmental stages of samples. **b**, mCG (left) and histone modification (right) signatures of primed distal feDMRs (blue; *n* = 618,786) and feDMRs (red; *n* = 3,715,052). The boxplots were generated using ‘boxplot’ function in R (3.3.1) and show the median and quartiles of the values in all non-liver tissues. Middle line, median; box, upper and lower quartiles; whiskers, 1.5 × (Q3 − Q1) above Q3 and below Q1; points, outliers. **c**, Number of enriched TF binding motifs only in feDMRs (red), only in primed distal feDMRs (orange), both (dark red) and none (grey). Only the motifs linked to expressed TFs (TPM ≥ 10) were included. Hypergeometric test was used to estimate the significance of overlap between motifs enriched in feDMRs and ones enriched in primed distal feDMRs. **d**, **e**, Similar to **a**, heatmaps showing the mCG levels of unxDMRs, including transposon overlapping unxDMRs (**d**) and non-transposon overlapping unxDMRs (**e**).

Notably, 76% of the CG-DMRs are more than 10 kb away from neighbouring transcription start sites (TSSs) (Extended Data Fig. 2b). Only 8.5% (*n* = 153,019) of CG-DMRs overlapped with promoters, CpG islands (CGIs) or CGI shores (Fig. 1d, Extended Data Fig. 2c–e). About 91.5% (1,655,791) of CG-DMRs were distally located and showed a high degree of evolutionary conservation, suggesting that they are functional (Fig. 1d, Extended Data Fig. 2f, g). By integrating these epigenomic data sets, we computationally delineated 468,141 CG-DMRs that are likely to be fetal enhancers (fetal enhancer-linked CG-DMRs or feDMRs) (see later section ‘Enhancer prediction with multi-omic data’; Supplementary Data). We further categorized the remaining CG-DMRs into four other types according to the degree of mCG difference and their relationship with transposons (Supplementary Note 2, Extended Data Figs. 2, 3). These results provided a comprehensive annotation of mCG variation throughout the mouse genome.

**Extended Data Fig. 4:**
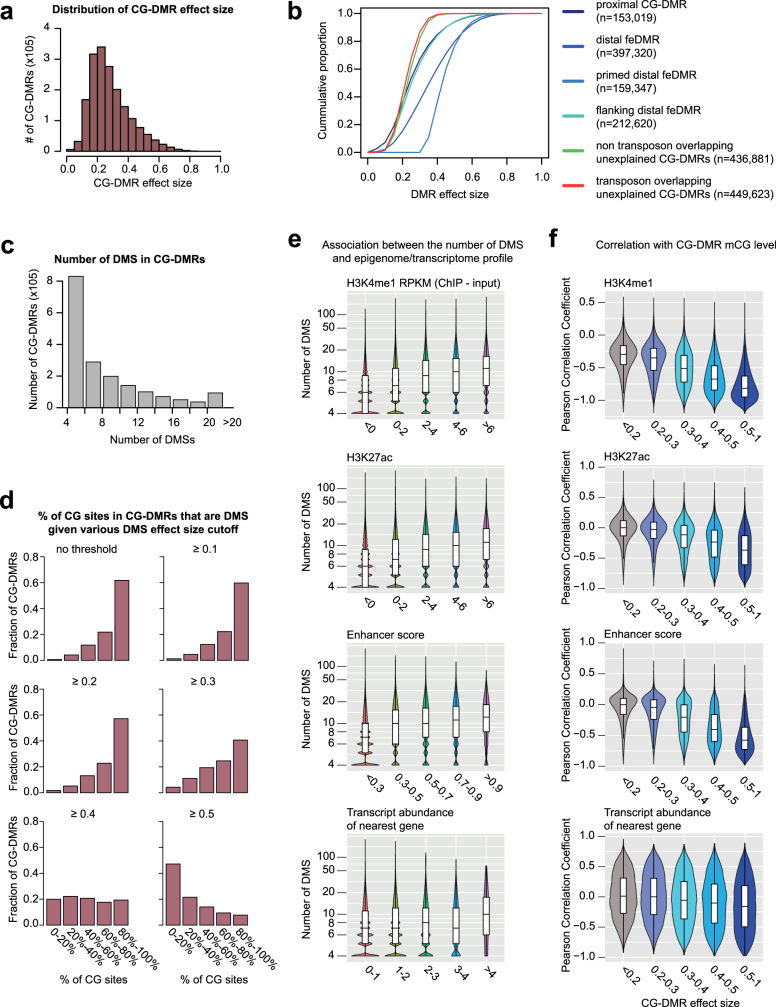
CG-DMR effect size analysis. **a**, Distribution of CG-DMR effect sizes. **b**, Cumulative distribution of CG-DMR effect sizes for CG-DMRs in different categories. **c**, Distribution of the number of DMSs in CG-DMRs. **d**, Fraction of CG sites in CG-DMRs that are DMSs given different DMS effect-size cutoffs. **e**, Number of DMSs in CG-DMRs with different H3K4me1 enrichment, H3K27ac enrichment, enhancer score (from REPTILE), and RNA abundance (log_10_(TPM + 1)) of the nearest genes with TSSs that are within 5 kb of CG-DMRs. The value was calculated in the most hypomethylated sample of each CG-DMR. The sample size for each violin or box from left to right is 732,389, 626,599, 254,925, 108,012 and 74,277 for H3K4me1, 935,017, 560,213, 136,778, 58,396 and 105,798 for H3K27ac, 1,593,822, 89,797, 70,254, 36,776 and 5,553 for enhancer score, and 1,045,863, 645,080, 98,020, 7,052 and 187 for gene expression. **f**, Distribution of Pearson correlation coefficient between mCG level and various metrics for CG-DMRs with different effect sizes. The metrics include H3K4me1 enrichment, H3K27ac enrichment, enhancer score (from REPTILE), and transcription (TPM) of nearest genes whose TSSs are within 5 kb of CG-DMRs. The number of CG-DMRs with effect size <0.2, 0.2–0.3, 0.3–0.4, 0.4–0.5 and 0.5–1 are 523,106, 615,414, 347,019, 184,116 and 138,512, respectively. Boxplots and violin plots were generated using ggplot2 (2.2.1) R (3.3.1) package. In the violin plot, width represents the density of different data values. In the boxplots, middle line, median; box, upper and lower quartiles; whiskers, 1.5 × (Q3 − Q1) above Q3 and below Q1; points, outliers.

The CG-DMRs show various degrees of difference in mCG level (effect size). The effect size of 71% of CG-DMRs is larger than 0.2, indicating that these CG-DMRs are present in at least 20% of cells in at least one tissue, while CG-DMRs in different categories showed distinct effect sizes (Extended Data Fig. 4a, b). On average, one CG-DMR contains 9 differentially methylated CG sites (DMSs), and in 62% of CG-DMRs, more than 80% of CG sites are DMSs (Extended Data Fig. 4c-d). CG-DMRs with more DMSs showed stronger predicted regulatory activity (Extended Data Fig. 4e). Similarly, as CG-DMRs with larger effect size are more likely to reflect bona fide mCG variation, they indeed showed stronger anti-correlation with active histone modifications and the transcription of nearby genes (Extended Data Fig. 4f, Supplementary Note 3).

We found some extensive changes in methylation near genes that are essential for fetal tissue development. For example, *Fabp7* is essential for establishing radial glial fibres in the developing brain^27^. In the forebrain, *Fabp7* underwent marked and continuous demethylation as the forebrain matured, associated with increased forebrain-specific acetylation at the 27th lysine residue of the H3 (H3K27ac) and *Fabp7* gene expression (Fig. 1e). In a different region, an experimentally validated enhancer (from VISTA enhancer browser^28^) of E11.5 heart, limb, nose and several other tissues, is hypomethylated in matched E11.5 tissue (Fig. 1f).

## Distinct pre- and postnatal mCG dynamics

**Fig. 2:**
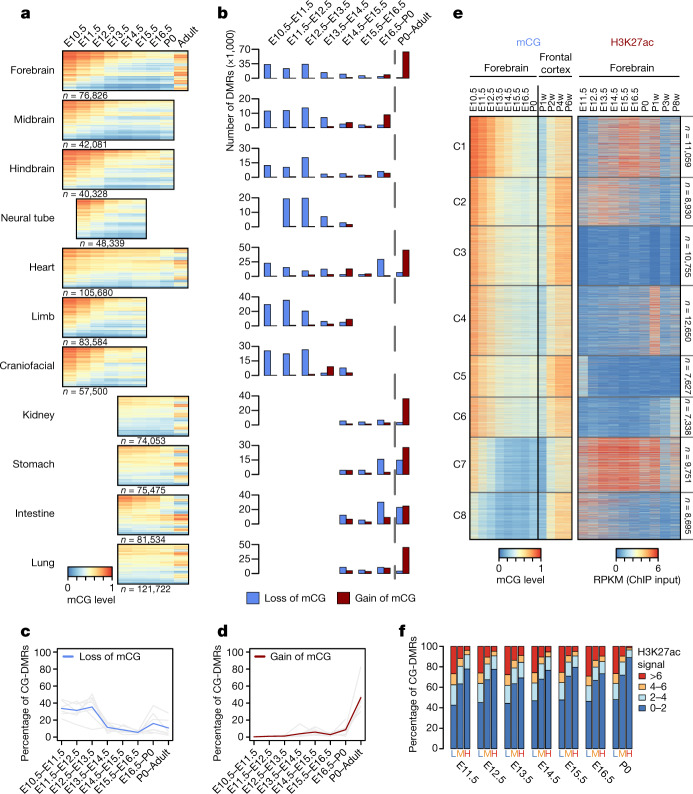
Tissue-specific CG-DMRs undergo continuous demethylation during embryogenesis and remethylation after birth. **a**, mCG levels of tissue-specific CG-DMRs. The adult forebrain was approximated using postnatal six-week-old frontal cortex^9^. Each row of the heatmaps represents an individual CG-DMR. **b**, The numbers of loss-of-mCG (blue) and gain-of-mCG (red) events in tissue-specific CG-DMRs for each developmental period (tissues aligned with **a**). **c**, **d**, Percentage of tissue-specific CG-DMRs that undergo loss of mCG (**c**) or gain of mCG (**d**) at each developmental period. Grey lines show the data for each non-liver tissue, and the blue or red line shows the mean. **e**, mCG and H3K27ac dynamics of forebrain-specific CG-DMRs. **f**, Relationship between mCG and H3K27ac in tissue-specific CG-DMRs. For each tissue type, tissue-specific CG-DMRs were grouped by their mCG level into low (L, mCG level ≤ 0.2), medium (M, 0.2 < mCG level ≤ 0.6) or high (H, mCG level > 0.6). Then, we quantified the fraction of tissue-specific CG-DMRs in each category that showed different levels of H3K27ac enrichment (Methods).

**Extended Data Fig. 5:**
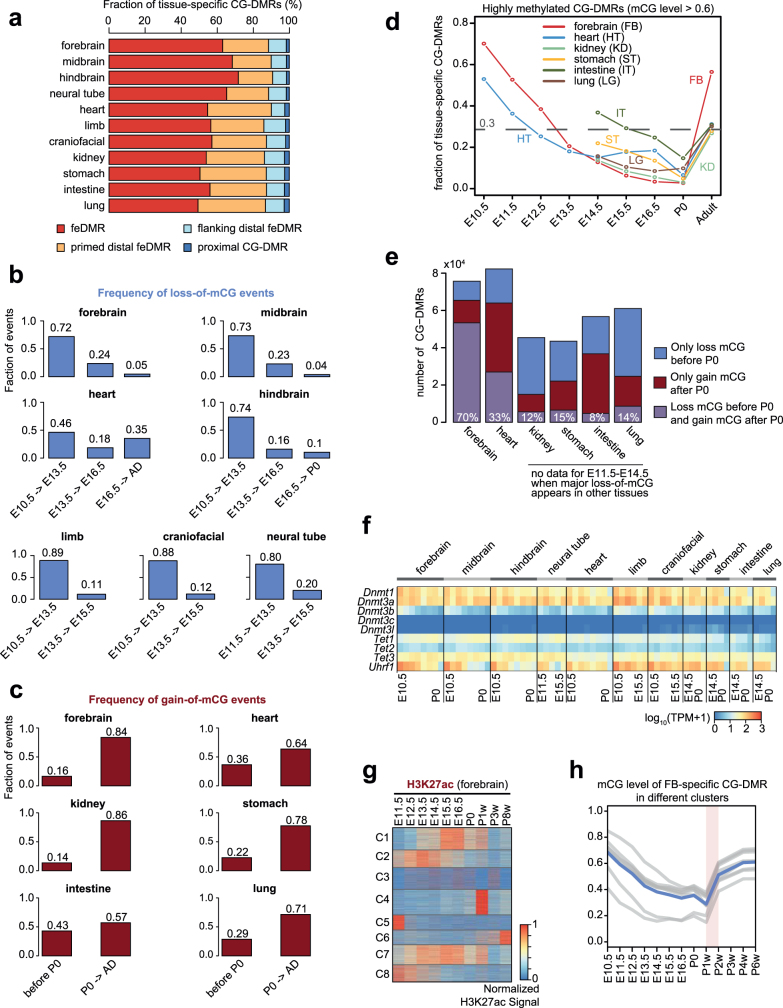
Link between methylation dynamics and histone modifications at tissue-specific CG-DMRs. **a**, Composition of tissue-specific CG-DMRs. **b**, **c**, Percentage of loss-of-mCG (**b**) and gain-of-mCG (**c**) events for different fetal stage intervals. **d**, Fraction of tissue-specific CG-DMRs that are heavily CG methylated (mCG level >0.6). **e**, Number and fraction of tissue-specific CG-DMRs that only gained mCG (mCG level increases by at least 0.1; red) after P0, only lost mCG (mCG level decreases by at least 0.1; blue), and both (purple) in six tissues for which adult methylome data are available. **f**, RNA abundance of genes involved in DNA methylation pathways, measured in TPM. **g**, Normalized H3K27ac signals in different clusters. **h**, Dynamic mCG level of forebrain-specific CG-DMRs. Grey lines, mean methylation levels of CG-DMRs in different clusters; blue line, mean of the mean of each cluster.

The dominant methylation pattern that emerged during fetal progression was a continuous loss of mCG at tissue-specific CG-DMRs, which overlap strongly with predicted enhancers (Fig. 2a, Extended Data Fig. 5a). This widespread demethylation is consistent with results from a previous study of whole mouse embryos^29^. By contrast, these CG-DMRs mainly gained mCG after birth (Fig. 2a). To quantify these changes for each developmental period, we counted loss-of-mCG and gain-of-mCG events (decreases or increases in mCG level of at least 0.1 in one CG-DMR) (Fig. 2b–d, Methods). From E10.5 to P0, 77–95% of the mCG changes were loss-of-mCG, more than 70% of which occurred between E10.5 and E13.5 in all tissues except heart (46%) (Extended Data Fig. 5b). The mCG level of 44–84% tissue-specific CG-DMRs dropped to below 0.5 at E14.5, compared to 16–31% at E10.5. As allele-specific methylation is relatively rare^8^, the observed methylation dynamics suggest that, at E14.5, most of the tissue-specific CG-DMRs are unmethylated in more than half of the cells in a tissue.

Compared to the loss of mCG, 57–86% of the gain-of-mCG events happened after birth (Extended Data Fig. 5c). As a result, 27–56% of the tissue-specific fetal CG-DMRs became highly methylated (mCG level >0.6) in adult tissues (at least 4 weeks old), compared to 0.3–15% at P0, which is likely to reflect the silencing of fetal regulatory elements (Extended Data Fig. 5d). In forebrain, 70% of forebrain-specific CG-DMRs underwent both prenatal loss-of-mCG and postnatal gain-of-mCG, coinciding with the marked methylomic reconfiguration during postnatal forebrain development^9^ (Extended Data Fig. 5e). However, only 33% of heart-specific CG-DMRs showed a similar trajectory, which might be associated with its relatively earlier maturation (Extended Data Fig. 5e). The percentage (8–15%) was even lower for CG-DMRs specific to kidney, lung, stomach and intestine, suggesting that major demethylation events are likely to occur during earlier developmental stages.

This widespread demethylation cannot be explained by the expression dynamics of the cytosine methytransferases *Dnmt1* and *Dnmt3a*, the co-factor *Uhrf1*^30^, or *Tet* methylcytosine dioxygenases, although a previous study^29^ reported the involvement of active DNA demethylation (Extended Data Fig. 5f). The absence of gain-of-mCG events until the postnatal period may involve translational and/or posttranslational regulation of these enzymes. Notably, WGBS does not distinguish between 5-methylcytosine and 5-hydroxymethylcytosine^31^, although earlier studies^9,32^ suggested that 5-hydroxymethylcytosines are relatively rare. Further studies that directly measure the full complement of cytosine modifications are needed to understand their dynamics during fetal tissue development.

## Linking dynamic mCG and chromatin states

To further pinpoint the timing of CG-DMR remethylation and its relationship with enhancer activity, we clustered forebrain-specific CG-DMRs on the basis of their mCG and H3K27ac dynamics across both fetal and adult stages (Fig. 2e, Extended Data Fig. 5g, Methods). In all clusters, mCG increased markedly between the first and second postnatal weeks and increased even further during tissue maturation in adult mice (Extended Data Fig. 5h).

We then investigated the association between mCG dynamics and predicted enhancer activity (approximated by H3K27ac abundance). Although depletion of mCG was not necessarily related to H3K27ac enrichment (for example, clusters 3, 5 and 6), high mCG was indicative of low H3K27ac (Fig. 2e, f). Only 2–9% of highly methylated CG-DMRs (mCG level >0.6) showed high H3K27ac enrichment (>6), whereas 25–28% of CG-DMRs with low methylation levels (mCG level <0.2) were enriched for H3K27ac (Fig. 2f). These observations suggest that decreases in cytosine methylation during fetal progression may precede and promote enhancer activity by increasing TF binding and/or altering histone modifications.

## Large-scale mCG features

**Extended Data Fig. 6:**
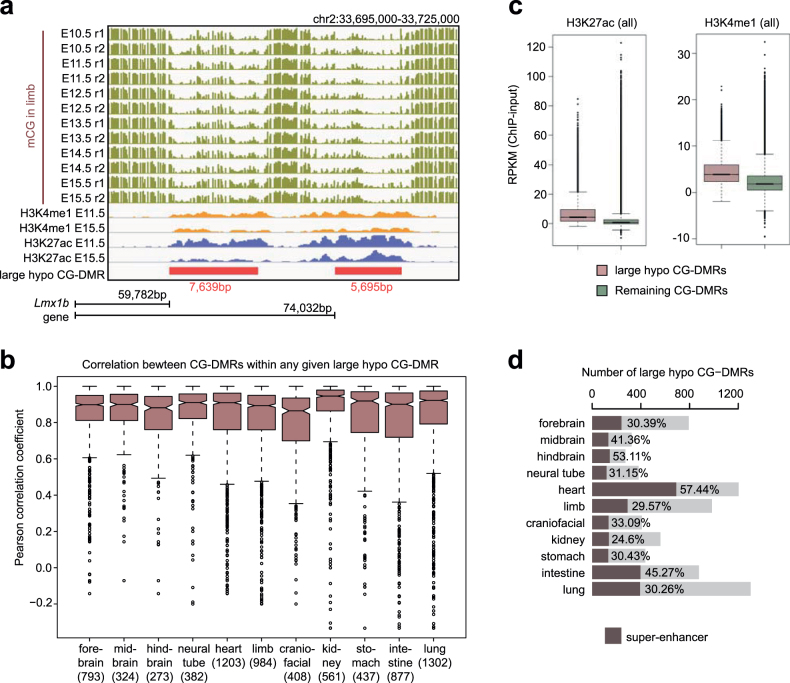
Large-scale CG hypomethylation overlaps strongly with super-enhancers. **a**, Epigenomic profiles of two limb-specific large hypo CG-DMRs near the *Lmx1* gene. Bottom, locations of the two large hypo CG-DMRs relative to *Lmx1*. **b**, Correlation between the mCG levels of CG-DMRs within the same large hypo CG-DMR across development stages in the given tissue type. If multiple CG-DMRs are within one large hypo CG-DMR, the mean Pearson correlation coefficient of all pairwise comparisons is reported. Numbers of CG-DMRs are shown in parentheses. **c**, H3K27ac and H3K4me1 enrichment in large hypo CG-DMRs (red; *n* = 39,729) and remaining CG-DMRs (green; *n* = 4,045,384) at all developmental stages across all tissue types except liver. **d**, Number of large hypo CG-DMRs identified in each tissue type and the percentage that overlap with super-enhancers (red). The boxplots (**b**, **c**) were generated using ‘boxplot’ function in R (3.3.1). Middle line, median; box, upper and lower quartiles; whiskers, 1.5 × (Q3 − Q1) above Q3 and below Q1; points, outliers.

In mouse neurons and a variety of human tissues, some CG-DMRs were found clustered together to form kilobase-scale hypomethylated domains, termed large hypo CG-DMRs^8,33^. We identified 273–1,302 such CG-DMRs in fetal tissues by merging adjacent CG-DMRs (Supplementary Table 3, Methods). For example, we found two limb-specific large hypo CG-DMRs upstream of *Lmx1b*, which is crucial for limb development^34^ (Extended Data Fig. 6a). The mCG levels of CG-DMRs within the same large hypo CG-DMR were well-correlated (average Pearson correlation coefficient 0.76–0.86) (Extended Data Fig. 6b). Compared with typical CG-DMRs, large hypo CG-DMRs showed higher levels of H3K4me1 and H3K27ac, while 25–57% of them overlapped with the putative super-enhancers^35,36^ defined by extremely high H3K27ac (Extended Data Fig. 6c, d, Methods). Similar to super-enhancers, the majority (58–79%) of large hypo CG-DMRs were intragenic (fold-enrichment 1.36–1.84, *P* < 0.001, Monte Carlo testing; Methods) and were associated with genes related to tissue functions (Supplementary Table 4).

**Extended Data Fig. 7:**
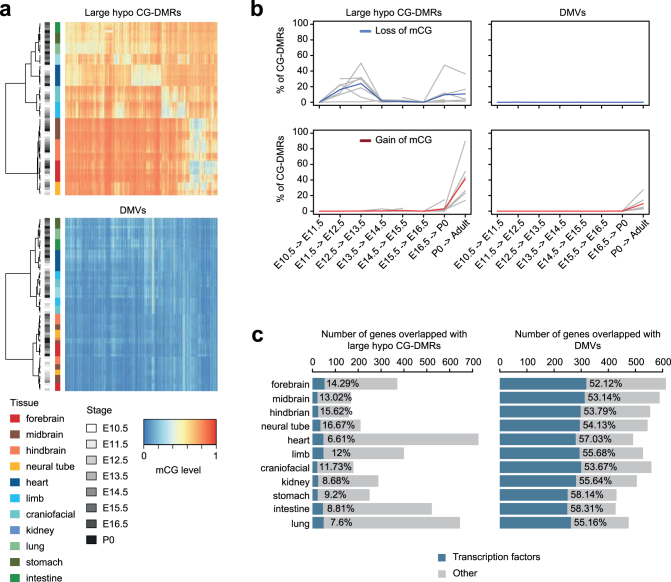
Comparing large hypo CG-DMRs and DMVs. **a**, mCG level of large hypo CG-DMRs (top) and DMVs (bottom) in all non-liver tissues. Both rows and columns were clustered using hierarchical clustering. Coloured bars indicate tissue types and grey bars indicate developmental stages of samples. The heatmap shows the data of merged large hypo CG-DMRs and DMVs for predictions from all tissue samples. **b**, Fraction of large hypo CG-DMRs (left) and DMVs (right) that undergo loss of mCG (top) and gain of mCG (bottom) during development. The blue (loss) or red (gain) line shows the aggregated values over all non-liver tissues, whereas the grey lines show the data for each tissue type. **c**, Number of genes that overlap with large hypo CG-DMRs (left) or DMVs (right). The dark blue bar indicates the number of genes that encode TFs.

We also found a different multi-kilobase DNA methylation feature called a DNA methylation valley or DMV^37,38^ (Supplementary Table 5, Methods). DMVs are ubiquitously unmethylated in all tissues across their developmental trajectory, whereas large hypo CG-DMRs display spatiotemporal hypomethylation patterns (Extended Data Fig. 7a, b). In fact, less than 4% of large hypo CG-DMRs overlapped with DMVs. Also, 53–58% of the DMV genes encode TFs, compared to 8–17% of genes in large hypo CG-DMRs (Extended Data Fig. 7c). The absence of repressive DNA methylation in DMVs implies that the expression of TF genes may be regulated by alternative mechanisms. Indeed, 510 out of 706 DMV genes (72.2%) are targets of the Polycomb repression complex^23^ (fold-enrichment 2.3, *P* < 0.001, hypergeometric test).

## mCH domains predict gene silencing

**Fig. 3:**
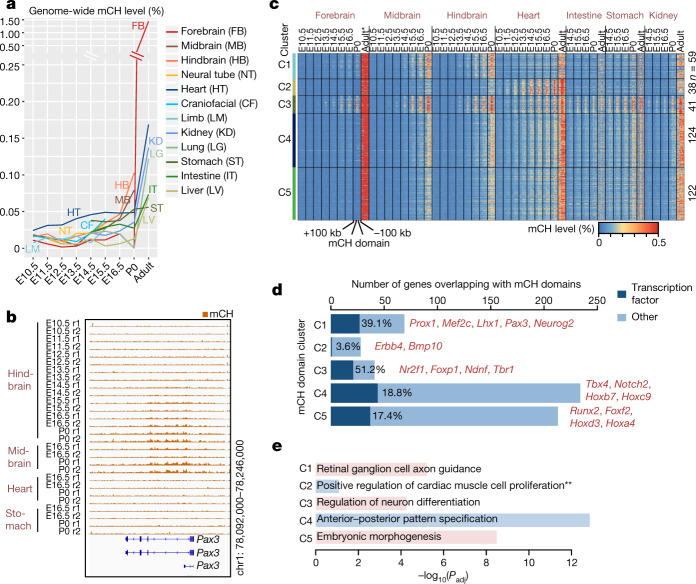
mCH accumulation predicts reduced gene expression. **a**, Global mCH level dynamics for each tissue. **b**, *Pax3*-overlapping mCH domain. **c**, mCH domain clustering based on mCH dynamics. *The adult forebrain was approximated using postnatal six-week-old frontal cortex^9^. **d**, mCH domain genes. Dark blue bars represent genes that encode TFs and examples are listed. **e**, The most enriched biological process terms from EnrichR^49^ for mCH domain genes. *P* values were calculated using one-tailed Fisher’s exact test with sample sizes of 69, 28, 41, 234 and 213 for C1, C2, C3, C4 and C5, respectively. *P* values were adjusted (*P*_adj_) for multiple testing correction using the Benjamini–Hochberg method. **P_adj_ = 0.082.

**Extended Data Fig. 8:**
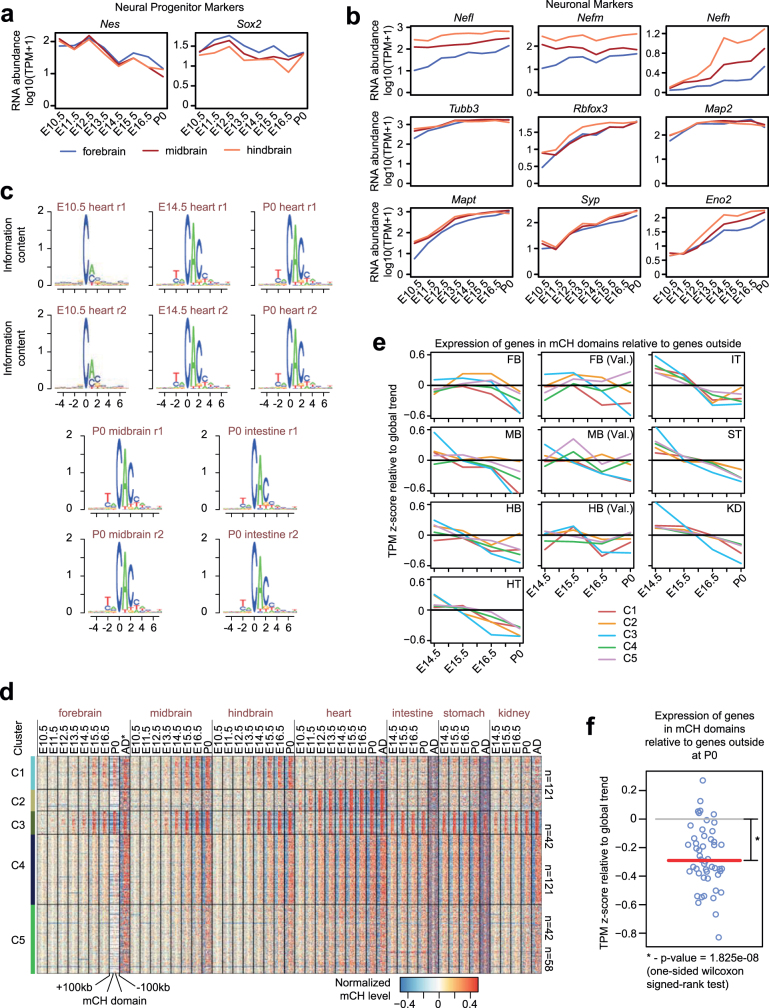
Non-CG methylation accumulation in fetal tissues. **a**, Expression of the neural progenitor marker genes *Nes*^39^ and *Sox2*^40^. **b**, Expression of several neuronal markers from ref. ^41^. **c**, Sequence context preference for non-CG methylation (mCH). **d**, Grouping mCH domains into five clusters according to the dynamics of methylation accumulation. The heatmap shows normalized methylation levels of mCH domains and flanking genomic regions (±100 kb). mCH in the adult (AD) forebrain was approximated using data from the frontal cortex from six-week-old mice. **e**, Expression dynamics of genes within mCH domains relative to the other genes. *Z*-scores were calculated for each gene across development and each line shows the mean value of mCH overlapping genes for each cluster. **f**, The expression of genes in mCH domains at P0 relative to the expression dynamics of genes outside mCH domains. Each circle corresponds to the value given one mCH domain cluster and one tissue. Red line indicates median, which was tested against 0 using a one-sided Wilcoxon signed-rank test (*n* = 50).

A less well-understood form of cytosine DNA methylation found in mammalian genomes is mCH^15^. mCH accumulates at detectable levels in nearly all tissues and organs during fetal progression (Fig. 3a). Notably, in brain tissues, the timing of mCH accumulation correlates with developmental maturation (downregulation of neural progenitor markers^39,40^ and upregulation of neuronal markers^41^) in sequential order of hindbrain, midbrain and forebrain (Fig. 3a, Extended Data Fig. 8a, b). Previous studies have shown that mCH is preferentially deposited at the 5′-CAG-3′ context in embryonic stem cells by DNMT3B and at 5′-CAC-3′ in adult tissues by DNMT3A^15^. In all fetal tissues, mCH is enriched at CAC sites and this specificity increases further as the tissues mature, implying a similar DNMT3A-dependent mCH pathway in both fetal and adult tissues (Extended Data Fig. 8c).

mCH accumulates preferentially at large genomic regions that we call ‘mCH domains’, which show higher mCH levels than their flanking sequences (Fig. 3b). We identified 384 mCH domains, which averaged 255 kb in length (Methods). Notably, 92% of them and 61% of their bases are intragenic (fold-enrichment 1.20 and 1.43, respectively; *P* < 0.001, Monte Carlo testing). Twenty-two per cent (128 out of 582) of the mCH domain genes (for example, *Pax3*) encode TFs, many of which are related to tissue development or organogenesis (fold-enrichment 3.23, *P* < 0.001, Monte Carlo testing).

To further explore the dynamics of mCH accumulation, we grouped mCH domains into five clusters, C1–C5 (Fig. 3b, c, Extended Data Fig. 8d, Methods). mCH domains in C1, C4 and C5 acquire mCH in all tissues (Fig. 3c). Notably, C1 is enriched for genes related to neuron differentiation, whereas C4 and C5 overlap with genes associated with embryo development (Fig. 3d, e, Supplementary Table 6). In contrast to these ubiquitous mCH domains, C2 gains mCH mostly in the heart, whereas C3 is brain-specific and overlaps with genes related to axon guidance (Fig. 3d, e).

As mCH accumulates in mCH domains during fetal progression, the mCH domain genes tend to be repressed compared to genes outside these domains, especially by P0 (Extended Data Fig. 8e, f). Because mCH domain genes are related to tissue, organ or embryo development, our data suggest that mCH is associated with silencing of the pathways of early fetal development. Notably, 382 of the 582 mCH domain genes are targeted by the Polycomb repressive complex pathway^23^ (fold-enrichment 2.0, *P* < 0.001, hypergeometric test). Consistent with our findings across fetal tissues, one study^42^ on postnatal brain reported that mCH acquired in gene bodies during postnatal brain development also repressed transcription. Further experiments, especially in the developing embryo, are necessary to delineate the mechanism of mCH regulation and its potential role in transcriptional regulation.

## Enhancer annotation based on multi-omic data

**Fig. 4:**
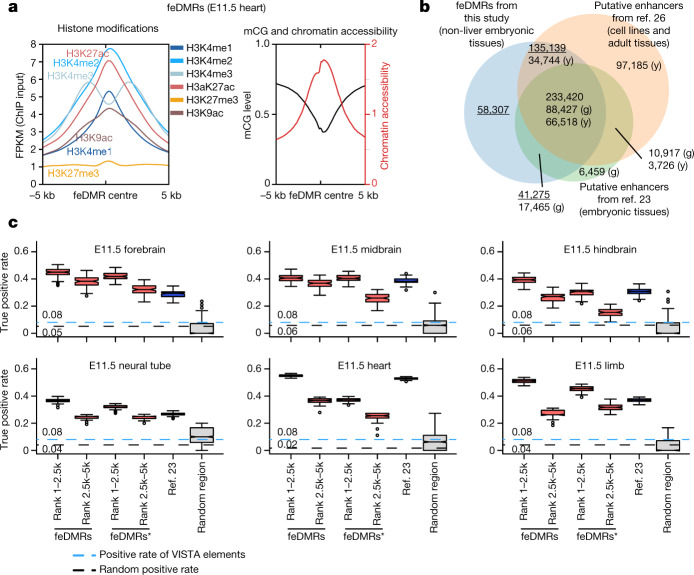
Enhancer annotation of developing mouse tissues. **a**, Chromatin signatures of feDMRs in E11.5 heart. The aggregate plots show the average histone modifications (left) and chromatin accessibility and mCG profiles (right) of ±5-kb regions flanking the feDMR centres. **b**, The overlap between feDMRs, adult enhancers from ref. ^26^, and putative enhancers from ref. ^23^. The letter in parenthesis indicates the enhancer set from which the number is calculated. g and y, putative enhancers from ref. ^23^ and ref. ^26^, respectively. Numbers related to feDMRs are underlined. **c**, True positive rate of putative enhancers on 100 down-sampled VISTA data sets in each E11.5 tissue for (from left to right): top 1–2,500 and 2,501–5,000 feDMRs; *top 1–2,500 and 2,501–5,000 feDMRs that do not overlap with the putative enhancers from ref. ^23^; top 1–2,500 putative enhancers from ref. ^23^ (blue); and random region (grey). The sample size is 1,000 for random region and 100 for all others. Random region indicates ten sets of randomly selected genomic regions with GC density and evolutionary conservation matching the top 5,000 feDMRs. Blue dashed line shows the fraction of elements that are experimentally validated enhancers (positives) in the dataset that is downsampled to match the estimated abundance of enhancers (see Supplementary Note 4 for details). Black dashed line indicates the random positive rate. Middle line, median; box, upper and lower quartiles; whiskers, 1.5 × (Q3 − Q1) above Q3 and below Q1; points, outliers.

To further investigate dynamic transcriptional regulation in developing fetal tissues, we predicted fetal CG-DMRs that are likely to be associated with enhancer activity using the REPTILE^43^ algorithm through the integration of mCG, histone modifications and chromatin accessibility profiles. We identified 468,141 candidate feDMRs (Methods, Supplementary Data). feDMRs show enhancer-like chromatin signatures, including open chromatin, depletion of mCG and H3K27me3, and enrichment for H3K4me1 and H3K27ac^7,25,44^ (Fig. 4a). Of the feDMRs identified, 99,582 (21.3%) have not previously been reported in adult mouse tissues^26^ and 58,307 (12.4%) were not captured by the chromatin state model (compared to the putative enhancers from ref. ^23^) (Fig. 4b).

**Extended Data Fig. 9:**
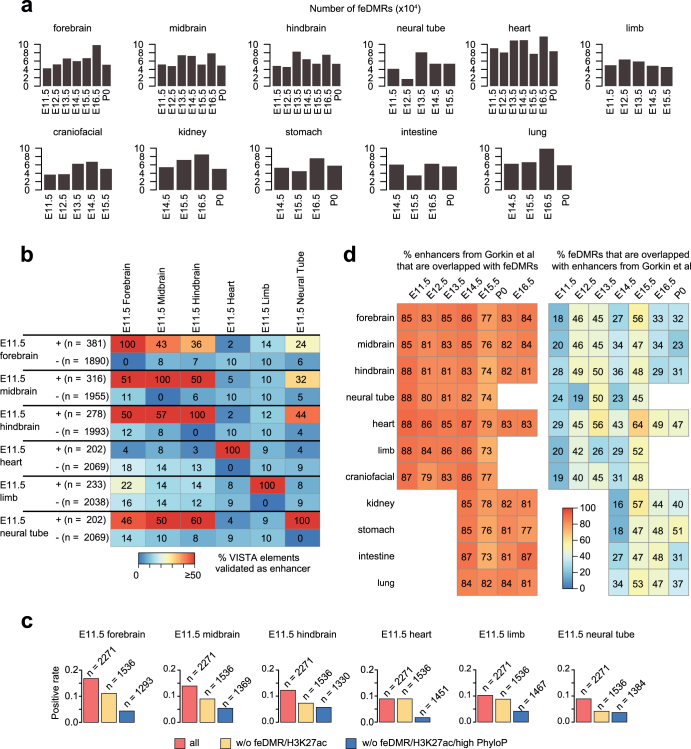
Fetal-enhancer-linked CG-DMRs. **a**, Number of feDMRs predicted in each tissue from each stage. **b**, Positive rate in E11.5 tissues of elements that were experimentally validated as an enhancer (+) or not (−) in a given E11.5 tissue. Numbers indicate the number of VISTA elements. **c**, Positive rate (fraction of elements that are experimentally validated as enhancer in a given tissue) of DNA elements in VISTA enhancer browser (left), ones that do not overlap with feDMRs or H3K27ac peaks (middle), and those that do not overlap with feDMRs or H3K27ac peaks and do not show a high evolutionary PhyloP score (right). Numbers in parentheses indicate the number of VISTA elements. **d**, Left, percentage of putative enhancers from ref. ^23^ that overlap with feDMRs in each tissue sample. Right, percentage of feDMRs that overlap with putative enhancers from ref. ^23^.

To evaluate the likelihood that these putative fetal enhancers are functional, we intersected feDMRs with VISTA enhancer browser DNA elements^28^, which were tested for enhancer activity by in vivo transgenic reporter assay in E11.5 mouse embryos. Even after carefully controlling for biases in the data set, 37–55% of the 2,500 (top 3–7%) most confident feDMRs that overlapped VISTA elements showed in vivo enhancer activity in matched tissues (Fig. 4c, Extended Data Fig. 9; Supplementary Note 4). Also, in any given tissue, feDMRs cover 73–88% of chromatin-state-based putative enhancers, and capture experimentally validated enhancers missing from the chromatin-state-based putative enhancers without compromising accuracy (Fig. 4c, Extended Data Fig. 9d). These results are consistent with previous findings that incorporating DNA methylation data improves enhancer prediction^43^. The validity of feDMRs is further supported by their evolutionary conservation, enrichment of TF binding motifs related to specific tissue function(s) and the enrichment of neighbouring genes in specific tissue-related pathways (Extended Data Fig. 2e–g, Supplementary Tables 7, 8, Methods).

## Linking mCG, enhancers and gene expression

**Fig. 5:**
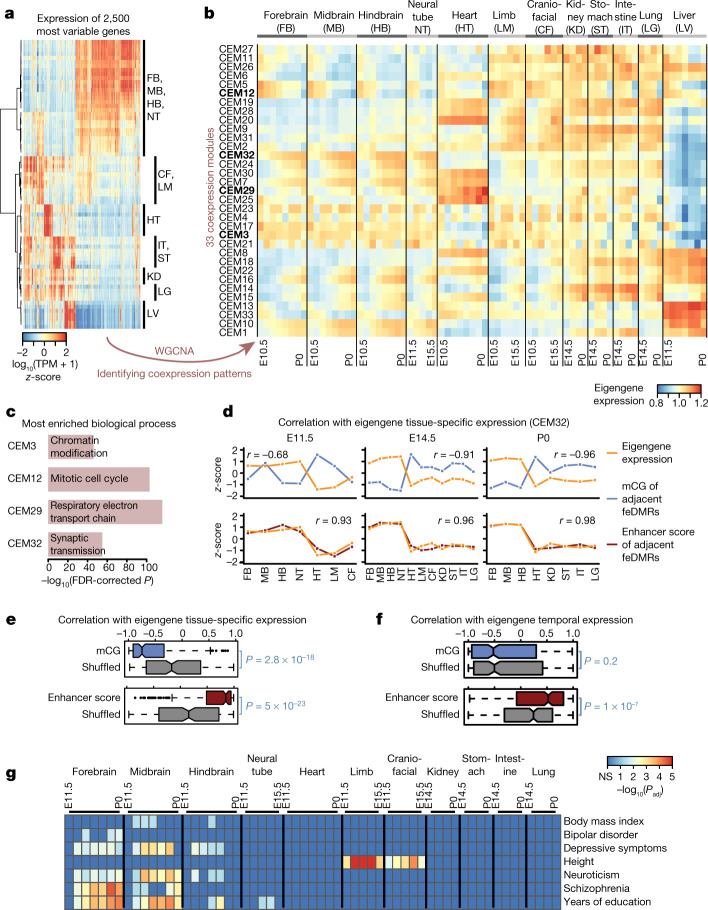
Association between mCG, gene expression and disease-associated SNPs. **a**, Expression profiles for 2,500 of the most variable genes. **b**, Thirty-three CEMs identified by WGCNA and their eigengene expression. CEMs shown in bold are related to **c**. **c**, The most enriched biological process terms of genes in four representative CEMs using EnrichR^49^. *P* values based on one-tailed Fisher’s exact test with sample sizes 6,766, 602, 126 and 2,968 for CEM3, CEM12, CEM29 and CEM32, respectively, adjusted for multiple testing correction using the Benjamini–Hochberg method. **d**, Correlation of the tissue-specific eigengene expression (orange) for each developmental stage with the mCG level or enhancer score (blue or red, respectively) *z*-scores of feDMRs linked to the genes in CEM32. Pearson correlation coefficients were calculated (*n* = 7, 11 and 8 for E11.5, E14.5 and P0, respectively). **e**, **f**, Pearson correlation coefficients of mCG level or enhancer score (blue or red, respectively) of feDMRs linked to the genes in each CEM with tissue-specific eigengene expression across all 33 CEMs on all stages (**e**), and temporal epigengene expression across all CEMs in all tissue types (**f**), excluding liver. *P* values based on two-tailed Mann–Whitney test (*n* = 231 (**e**), *n* = 363 (**f**)). Middle line, median; box, upper and lower quartiles; whiskers, 1.5 × (Q3 − Q1) above Q3 and below Q1; points, outliers. **g**, feDMRs are enriched for human GWAS SNPs associated with tissue- or organ-specific functions and tissue-related disease states. *P* values calculated using LD score regression^47^, adjusted for multiple testing correction using the Benjamini–Hochberg approach.

**Extended Data Fig. 10:**
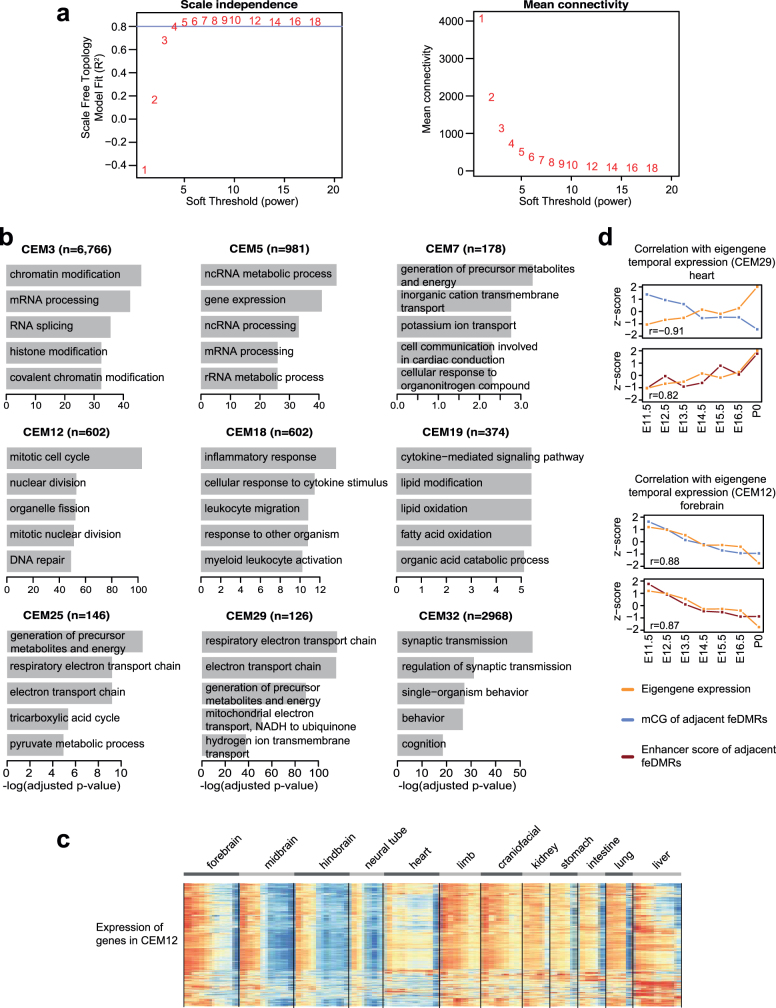
WGCNA identification of co-expression modules. **a**, The scale-free topology model fit (*R*^2^) (top) and the mean connectivity of the coexpression network (bottom) given different soft-thresholding powers. These two plots show how thresholds were chosen for WGCNA. Blue line indicates model fit cutoff (*R*^2^ = 0.8). A soft threshold of 5 was chosen to construct the co-expression network because it is the first threshold value at which the model fit is greater than 0.8. **b**, Top enriched ontology terms of genes in co-expression modules. EnrichR^49,77^ was used for this analysis, which uses a one-tailed Fisher’s exact test to calculate *P* values (number of overlapping genes shown in parentheses). The Benjamini–Hochberg method was used to adjust *P* values for multiple testing correction. **c**, Expression of genes in CEM12. Each row is a gene in certain module and the TPM *z*-scores were calculated along each row. **d**, Similar to Fig. 5d, correlation of temporal eigengene expression for CEM29 (top) and CEM12 (bottom) with the *z*-scores of average mCG level (top plots) and the *z*-scores of average enhancer score of neighbouring feDMRs (bottom plots) (Pearson correlation coefficient; *n* = 7).

Finally, we investigated the association of mCG dynamics with the expression of genes in different biological processes or pathways. Using weighted correlation network analysis (WGCNA)^45^, we identified 33 clusters of co-expressed genes (co-expression modules, CEMs) and calculated ‘eigengenes’ to summarize the expression profile of genes within modules (Fig. 5a, b, Extended Data Fig. 10a, Methods). Genes that share similar expression profiles are more likely to be regulated by a common mechanism and/or to be involved in the same pathway (Extended Data Fig. 10b, Supplementary Table 9). For example, genes in CEM12, which are related to cell cycle, are highly expressed in early developmental stages but are downregulated as tissues mature, matching our knowledge that cells become post-mitotic in mature tissues (Fig. 5c, Extended Data Fig. 10c).

To understand how mCG and the enhancer activity of feDMRs are associated with the expression of genes in CEMs, we linked feDMRs to their neighbouring genes. Then, we correlated the eigengene expression of each CEM with the average mCG levels (or enhancer score) of feDMRs linked to the genes in that CEM (Methods). To tease out tissue-specific and temporal associations, we calculated the correlation across tissues and across developmental stages separately. Across all tissue samples from a given developmental stage, mCG of feDMRs was negatively correlated with eigengene expression, whereas enhancer score was positively correlated with eigengene expression (Fig. 5d, e). We then calculated the correlation across samples of a given tissue type from different developmental stages. Whereas mCG levels generally decreased at feDMRs over development (Fig. 2a), the enhancer score remained positively correlated with temporal expression (Fig. 5f, Extended Data Fig. 10d). These results imply that feDMRs are likely to drive both tissue-specific and temporal gene expression.

## Genetic risk factors enriched in feDMRs

The vast majority of genetic variants associated with human diseases that have been identified in genome-wide association studies (GWAS) are located in non-coding regions. These non-coding variants, as well as the heritability of human diseases, are enriched in the distal regulatory elements of related tissues and cell types^46,47^. The spatiotemporal mouse enhancer activity annotation (feDMRs) and the degree of evolutionary conservation in regulatory elements between human and mouse^26^ make it possible to analyse disease- or trait-associated loci, and to pinpoint the related tissue(s) and developmental time point(s) in the mouse ENCODE data. To do this, we applied stratified linkage disequilibrium (LD) score regression^47^ to partition the heritability of 27 traits in the human orthologous regions of the mouse feDMRs (Methods). We found that the heritability of human disease- and trait-associated single-nucleotide polymorphisms (SNPs) was significantly enriched in the orthologous regions of mouse feDMRs for each corresponding tissue (Fig. 5g, Supplementary Table 10; LD score regression^47^ (Methods)). For example, the heritability of schizophrenia and ‘years of education’ is enriched in forebrain- and midbrain-specific feDMRs, whereas craniofacial- and limb-specific feDMRs are enriched for the heritability of height (Fig. 5g). Some associations between traits or diseases and tissue-specific feDMRs were found only at certain developmental stages (Fig. 5g). For example, schizophrenia loci are associated with forebrain feDMRs only at E12.5–P0. Similar results were also found at human orthologues of regions that showed spatiotemporal differences in open chromatin^23^. Given current challenges in obtaining human fetal tissue, our results suggest that it might be possible to integrate human genetic data with fetal spatiotemporal epigenomic data from model organisms to predict the relevant tissue or organ type(s) for a variety of human developmental diseases.

## Discussion

We have described the generation and analysis of a comprehensive collection of base-resolution, genome-wide maps of cytosine DNA methylation for twelve tissues and organs from eight distinct developmental stages of mouse embryogenesis and the adult stage. By integrating DNA methylation with histone modification, chromatin accessibility and RNA-seq data from the same tissue samples from companion papers^23,24^, we have annotated 1,808,810 methylation-variable genomic elements, encompassing nearly a quarter (613 Mb) of the mouse genome and generating predictions for 468,141 fetal enhancer elements. The counterparts of these fetal enhancers in the human genome are tissue-specifically enriched for genetic risk loci associated with a variety of developmental disorders or diseases. Such enrichments suggest that it might be possible to generate new mouse models of human disease by introducing the candidate disease-associated alleles into feDMRs using genome-editing techniques^48^.

The temporal nature of these data sets enabled us to uncover simple mCG dynamics at predicted DNA regulatory regions. During early stages of fetal development, methylation decreases at predicted fetal regulatory elements in all tissues until birth, after which time it rises markedly. As the tissues that we have investigated comprise a variety of cell types, a fraction of the observed dynamics might result from changes in DNA methylation during the differentiation of individual cell types and/or the changing cell type composition during development. In spite of the tissue heterogeneity, such dynamics suggest a plausible regulatory principle in which metastable repressive mCG is removed to enable more rapid, flexible modes of gene regulation (for example, histone modification or changes in chromatin accessibility).

In addition, our findings extend current knowledge of non-CG methylation, an understudied context of cytosine modification. During fetal development, there is preferential accumulation of mCH in specific tissues at genomic locations, each hundreds of kilobases in size. We call these genomic features ‘mCH domains’. Genes that lie in mCH domains are downregulated in their expression as mCH further accumulates during the later stages of fetal development. Although its function remains debatable, in vivo and in vitro studies indicate that mCH directly increases the binding affinity of MeCP2^18^, which is highly expressed in the brain and mutation of which leads to Rett syndrome. Gene-rich mCH domains in non-brain tissues are likely to be enriched for undiscovered mCH binding proteins, which, as with MeCP2, may be involved in recruiting transcriptional repressor complexes and thereby promoting gene repression.

Despite the broad scope of this study, it is important to note its limitations. First, several tissues, such as skeleton, gonads and pancreas, were not included in the data set. Also, sex-related differences were not studied. In addition, the tissues examined in this study are heterogeneous, and thus future efforts to examine the epigenomes of individual cells will be critical for a deeper understanding of the gene regulatory programs.

Overall, we present, to our knowledge, the most comprehensive set of temporal fetal tissue epigenome mapping data available in terms of the number of developmental stages and tissue types investigated, expanding upon the previous phase of the mouse ENCODE project^26^, which focused exclusively on adult mouse tissues. Our results highlight the power of this data set for analysing regulatory element dynamics in fetal tissues during in utero development. These spatiotemporal epigenomic data sets provide a valuable resource for answering fundamental questions about gene regulation during mammalian tissue and organ development as well as the possible origins of human developmental diseases.

## Methods

### Tissue collection

All animal work was reviewed and approved by the Lawrence Berkeley National Laboratory Animal Welfare and Research Committee or the University of California, Davis Institutional Animal Care and Use Committee.

Mouse fetal tissues were dissected from embryos of different developmental stages from female C57Bl/6N *Mus musculus*. Mice used for obtaining tissue samples at E14.5 and P0 were purchased from Charles River Laboratories (C57BL/6NCrl strain) and Taconic Biosciences (C57BL/6NTac strain). Mice used for obtaining tissue samples at remaining developmental stages were purchased from Charles River Laboratories (C57BL/6NCrl strain). The number of embryos or P0 pups collected was determined by whether the materials were sufficient for genomic assay, and was not based on statistical considerations. Between 15 and 120 embryos or pups were collected for each replicate of each tissue at each stage.

### Tissue excision and fixation

See Supplementary Files 1, 2 for details.

### MethylC-seq library construction and sequencing

MethyC-seq libraries were constructed as previously described^8^ and a detailed protocol is available^50^. An Illumina HiSeq 2500 system was used for all WGBS using either 100- or 130-base single-ended reads.

### Mouse reference genome construction

For all analyses in this study, we used mm10 as the reference genome, which includes 19 autosomes and two sex chromosomes (corresponding to the’mm10-minimal’ reference in the ENCODE portal, https://www.encodeproject.org/). The fasta files of mm10 were downloaded from the UCSC genome browser (9 June 2013)^51^.

### WGBS data processing

All WGBS data were mapped to the mm10 mouse reference genome as previously described^52^. WGBS processing includes mapping of the bisulfite-treated phage lambda genome spike-in as control to estimate the sodium bisulfite non-conversion rate. This pipeline (called methylpy) is available on github (https://github.com/yupenghe/methylpy). In brief, cytosines within WGBS reads were first computationally converted to thymines. The converted reads were then aligned by bowtie (1.0.0) onto the forward strand of the C–T converted reference genome and the reversed strand of the G–A converted reference genome, separately. We filtered out reads that were not uniquely mapped or were mapped to both computationally converted genomes. Next, PCR duplicate reads were removed. Last, methylpy counted the methylated basecalls (cytosines) and unmethylated basecalls (thymines) for each cytosine position in the corresponding reference genome sequence (mm10 or lambda).

### Calculation of methylation level

Methylation level was computed to measure the intensity and degree of DNA methylation of single cytosines or larger genomic regions. The methylation level is defined as the ratio of the sum of methylated basecall counts over the sum of both methylated and unmethylated basecall counts at one cytosine or across sites in a given region^53^, subtracting the sodium bisulfite non-conversion rate. The sodium bisulfite non-conversion rate is defined as the methylation level of the bisulfite-treated lambda genome.

We calculated this metric for cytosines in both CG context and CH contexts (H = A, C or T). The former is called the CG methylation (mCG) level or mCG level and the latter is called the CH methylation (mCH) level or mCH level.

### Quality control of WGBS data

We calculated several quality control metrics for all the WGBS data and the results are presented in Supplementary Table 1. For each tissue sample, we calculated cytosine coverage, sodium bisulfite conversion rate, and reproducibility between biological replicates. Cytosine coverage is the average number of reads that cover cytosine. In the calculation, we combined the data of both strands. Sodium bisulfite conversion rate measures the sodium bisulfite conversion efficiency and is calculated as one minus the methylation level of unmethylated lambda genome. The reproducibility of biological replicates is defined as the Pearson correlation coefficient of mCG quantification between biological replicates for sites covered by at least ten reads.

All of the WGBS data passed ENCODE standards (https://www.encodeproject.org/data-standards/wgbs/) and are accepted by the ENCODE consortium. Almost all of the biological replicates of tissue samples have at least 30× cytosine coverage. All biological replicates have at least 99.5% sodium bisulfite conversion rate. All non-liver tissue samples have reproducibility greater than 0.8. The reproducibility of liver samples is slightly lower but is still greater than 0.7. The reduced reproducibility is due to the increase in sampling variation, which is a result of genome-wide hypomethylation in the liver genome.

### ChIP–seq data processing

ChIP–seq data were processed using the ENCODE uniform processing pipeline for ChIP-seq. In brief, Illumina reads were first mapped to the mm10 reference using bwa^54^ (version 0.7.10) with parameters ‘-q 5 -l 32 -k 2’. Next, the Picard tool (http://broadinstitute.github.io/picard/, version 1.92) was used to remove PCR duplicates using the following parameters: ‘REMOVE_DUPLICATES=true’.

We represented each histone modification mark as continuous enrichment values of 100-bp bins across the genome. The enrichment was defined as the RPKM after subtracting ChIP input. The enrichment across the genome was calculated using bamCompare in Deeptools2^55^ (2.3.1) using options ‘–binSize 100–normalizeUsingRPKM–extendReads 300–ratio subtract’. For the ChIP–seq data of the transcriptional co-activator EP300 (E1A-associated protein p300), we used MACS^56^ (1.4.2) to call peaks using default parameters.

### RNA-seq data

Processed RNA-seq data for all fetal tissues from all stages were downloaded from the ENCODE portal (https://www.encodeproject.org/; Supplementary Table 2).

To further validate our findings regarding transcriptomes generated across the Wold and Ecker laboratories, we generated an additional two replicates of RNA-seq data for fetal forebrain, midbrain, hindbrain and liver tissues. We first extracted total RNA using the RNeasy Lipid tissue mini kit from Qiagen (cat no. 74804). Then, we used the Truseq Stranded mRNA LT kit (Illumina, RS-122-2101 and RS-122-2102) to construct stranded RNA-seq libraries on 4 μg of the extracted total RNA. An Illumina HiSeq 2500 was used to sequence the libraries and generate 130-base single-ended reads.

### RNA-seq data processing and gene expression quantification

RNA-seq data were processed using the ENCODE RNA-seq uniform processing pipeline. In brief, RNA-seq reads were mapped to the mm10 mouse reference using STAR^57^ aligner (version 2.4.0k) with GENCODE M4 annotation^58^. We quantified gene expression levels using RSEM (version 1.2.23)^59^, expressed as TPM. For all downstream analyses, we filtered out non-expressed genes and only retained genes that showed non-zero TPM in at least 10% of samples.

### ATAC–seq data

ATAC–seq data for all fetal tissues from all stages were downloaded from the ENCODE portal (https://www.encodeproject.org/; Supplementary Table 2). ATAC–seq reads were mapped to the mm10 genome using bowtie (1.1.2) with flag ‘-X 2000–no-mixed–no-discordant’. Then, we removed PCR duplicates using samtools^60^ and mitochondrial reads. Next, we converted read ends to account for Tn5 insertion position by moving the read end position by 4 bp towards the centre of the fragment. We converted paired-end read ends to single-ended read ends. Last, we used MACS2 (2.1.1.20160309) with flags ‘—nomodel —shift 37 —ext 73 —pval 1e-2 -B —SPMR —call-sumits’ to generate signal track files in bigwig format. MACS2 calculated ATAC–seq read fold enrichment over the background MACS2 moving window model. This fold enrichment is used as the intensity/signal of chromatin accessibility.

### Genomic features of mouse reference genome

We used GENCODE M4^58^ gene annotation in this study. CGI annotation was downloaded from UCSC genome browser (5 September 2016)^51^. CGI shores are defined as the upstream 2 kb and downstream 2 kb regions along CGIs. Promoters are defined as regions from −2.5 kb to +2.5 kb around TSSs. CGI promoters are defined as those that overlap with CGIs while the remaining promoters are called non-CGI promoters.

We also obtained a list of mappable transposable elements (TEs) using the following procedure. RepeatMasker annotation of the mm10 mouse genome was downloaded from UCSC genome browser (12 September 2016)^51^. The annotation included 5,138,231 repeats. We acquired the transposon annotation by selecting only repeats that belonged to one of the following repeat classes (repClass): ‘DNA’, ‘SINE’, ‘LTR’ or ‘LINE’. Then, we excluded any repeat elements with a question mark in their name (repName), class (repClass) or family (repFamily). For the remaining 3,643,962 transposons, we further filtered out elements that contained fewer than two CG sites or cases within which less than 60% of CG sites were covered by at least ten reads across all samples when the data from two replicates were combined. Finally, we used the remaining set of 1,688,189 mappable transposons for analyses in this study.

### CG-DMRs

We identified CG-DMRs using methylpy (https://github.com/yupenghe/methylpy) as previously described^52^. In brief, we first called DMSs and then merged them into blocks if they both showed similar sample-specific methylation patterns and were within 250 bp. Last, we filtered out blocks containing fewer than three DMSs. In this procedure, we combined the data from the two biological replicates for all tissues, excluding liver samples owing to global hypomethylation of the genome.

We overlapped the resulting fetal tissue CG-DMRs with CG-DMRs previously identified^11^ using ‘intersectBed’ from bedtools^61^ (v2.27.1). The mm9 coordinates of the CG-DMRs from ref. ^11^ were first mapped to mm10 using liftOver^51^ with default parameters. Overlap of CG-DMRs is defined as a CG-DMR with at least one base overlap with another CG-DMR when comparing genomic coordinates between lists.

### Identification of tissue-specific CG-DMRs

For each fetal tissue type, we defined tissue-specific CG-DMRs as those that showed hypomethylation in a tissue sample from any fetal stage (E10.5 to P0). Hypomethylation is meaningful only with a baseline, thus we used an outlier detection algorithm^62^ to defined the baseline mCG level of each CG-DMR across tissue samples using the mean of the bulk, which was defined as the value for the narrowest mCG level range that includes half of all samples. Specifically, ${x}_{s}^{i}$ is the mCG level of CG-DMR *i* (*i* = 1,…,*M*) in tissue sample *s* (*s* = 1,…,*N*). Assuming the samples are ordered such that ${x}_{1}^{i}\le {x}_{2}^{i}\ldots \le {x}_{s}^{i}\ldots \le {x}_{N}^{i}$, the baseline is defined as ${b}_{i}=\frac{1}{\lceil N/2\rceil }{\sum }_{s=a+1}^{a+\lceil N/2\rceil }{x}_{s}^{i}$, in which *a* is the sample index such that ${x}_{a+\lceil N/2\rceil }^{i}-{x}_{a+1}^{i}$ is minimized, that is, $a=\text{arg}\,\mathop{min}\limits_{t}({x}_{t+\lceil N/2\rceil }^{i}-{x}_{t+1}^{i})$. $\lceil N/2\rceil$ is defined as the smallest integer that is greater than or equal to *N*/2. Last, we defined hypomethylated samples as samples in which the mCG level at CG-DMR *i* is at least 0.3 smaller than baseline *b*_*i*_, that is, $\{s|({x}_{s}^{i}-{b}_{i})\le -0.3\}$. Then, CG-DMR *i* is specific to these tissues. Liver data were not included in this analysis and we excluded CG-DMRs that had zero coverage in any of the non-liver samples. In total, only 402 CG-DMRs (about 0.02%) were filtered out.

### Linking CG-DMRs with genes

We linked CG-DMRs to their putative target genes on the basis of genomic distance. First, we only considered expressed genes that showed non-zero TPM in at least 10% of all fetal tissue samples. Next, we obtained coordinates for TSSs of the expressed genes and paired each CG-DMR with the closest TSS using ‘closestBed’ from bedtools^61^. In this way, we inferred a target gene for each CG-DMR; these gene–TSS associations were used in all subsequent analyses in this study.

### Predicting feDMRs

The REPTILE^43^ algorithm was used to identify the CG-DMRs that showed enhancer-like chromatin signatures. We called these feDMRs. REPTILE uses a random forest classifier to learn and then distinguish the epigenomic signatures of enhancers and genomic background. One unique feature of REPTILE is that by incorporating the data of additional samples (as outgroup/reference), it can use epigenomic variation information to improve enhancer prediction. In this study, REPTILE was run using input data from CG methylation (mCG), chromatin accessibility (ATAC–seq) and six histone marks (H3K4me1, H3K4me2, H3K4me3, H3K27ac, H3K27me3 and H3K9ac).

A REPTILE enhancer model was trained in similar way previously^43^. In brief, CG-DMRs were called across the methylomes of mouse embryonic stem cells (mES cells) and all eight E11.5 mouse tissues. CG-DMRs were required to contain at least two DMSs and they were extended 150 bp in each direction (5′ and 3′). The REPTILE model was trained on the mES cell data using E11.5 mouse tissues as an outgroup. Data from mCG and six histone modifications are available for these samples. The training data set consists of 5,000 positive instances (putative known enhancers) and 35,000 negative instances. Positives were 2-kb regions centred at the summits of the top 5,000 EP300 peaks in mES cells. Negatives include 5,000 randomly chosen promoters and 30,000 randomly chosen 2-kb genomic bins. The bins have no overlap with any positives or promoters. REPTILE learned the chromatin signatures that distinguish positive instances from negative instances.

Next, using this enhancer model, we applied REPTILE to delineate feDMRs from the 1,808,810 CG-DMRs identified across all non-liver tissues. feDMRs were predicted for each sample based on data from mCG and six core histone marks, while the remaining non-liver samples were used as an outgroup. In REPTILE, the random forest classifier for CG-DMR assigns a confidence score ranging from 0.0 to 1.0 to each CG-DMR in each sample. This score corresponds to the fraction of decision trees in the random forest model that vote in favour of the CG-DMR being an enhancer. Previous benchmarks showed that the higher the score, the more likely it was that a CG-DMR shows enhancer activity^43^. We named this confidence score the enhancer score. For each tissue sample, feDMRs are defined as CG-DMRs with an enhancer score greater than 0.3. feDMRs were also defined for each tissue type as the CG-DMRs that were identified as an feDMR in at least one tissue sample of that tissue type. For example, if a CG-DMR was predicted as an feDMR only in E14.5 forebrain, it was classified as a forebrain-specific feDMR.

We overlapped the feDMRs with putative adult enhancers from ref. ^26^. We used a set of coordinates to identify the centre base position of putative enhancers for each of the tissues and cell types from http://mouseencode.org/publications/mcp00/. Next, we defined putative enhancers as ±1-kb regions around the centres. Putative enhancers from different tissues and cell types were combined and merged if they overlapped. The merged putative enhancers (mm9) were then mapped to the mm10 reference using liftOver^51^. Finally, ‘intersectBed’ from bedtools^61^ was used to overlap feDMRs with these putative enhancers.

### Evaluating feDMRs with experimentally validated enhancers

We used enhancer data from the VISTA enhancer browser^28^ to estimate the fraction of feDMRs that display enhancer activity in vivo. Specifically, we calculated the fraction of feDMR-overlapping VISTA elements that have been experimentally validated as enhancers, which we termed the true positive rate. We evaluated the true positive rate of feDMRs for six E11.5 tissues (forebrain, midbrain, hindbrain, heart, limb and neural tube), where at least 30 VISTA elements had been experimentally validated as enhancers (positives).

However, the selection of the VISTA elements was biased. Compared to randomly selected sequences, they are more enriched for enhancers, which will lead to an overestimate of the true positive rate. To reduce the effect of selection bias, we needed to first estimate the fraction of VISTA elements that are positives (positive rate) in a given tissue if there is minimal selection bias. We termed this fraction the genuine positive rate. Details can be found in Supplementary Note 4. Then, we can sample the current VISTA data set to construct data sets with a positive rate that matches the genuine positive rate. As the positive rate is not inflated in the constructed data sets, it will allow a fair evaluation of our enhancer prediction approach (also see Supplementary Note 4 for details).

Using the bias-controlled data sets, we calculated the true positive rate of feDMRs for each E11.5 tissue. First, we ranked feDMRs by their enhancer scores (from highest to lowest). We then overlapped the top 2,500 (or top 2,501–5,000) feDMRs of a given E11.5 tissue with VISTA elements, requiring that at least one feDMR is fully contained for a VISTA element to be counted as overlapped. Last, we calculated the fraction of feDMR-overlapping VISTA elements that are experimentally validated enhancers in the given tissue (that is, the true positive rate).

To better interpret the true positive rate of feDMRs, we also evaluated 5,000 randomly selected genomic bins with GC content and degree of evolution conservation (PhyloP score) matching the top 5,000 feDMRs. We used this method as a baseline. For each E11.5 tissue, we repeated this random selection process ten times and generated ten sets of random regions. Next, we calculated the true positive rate of each set of random regions in the bias-controlled data sets. As an additional baseline method, we also calculated the positive rate of VISTA elements that did not overlap with any feDMRs or H3K27ac peaks.

### Comparing feDMRs with putative enhancers based on chromatin state

Chromatin state-based putative enhancers are genomic regions labelled as enhancer states (states 5, 6 and 7) by ChromHMM^63^ in non-liver tissue samples (ref. ^23^). To fairly compare their validation rate with that of feDMRs, we needed to select the top 2,500 putative enhancers. ChromHMM does not assign a score and therefore we instead ranked these elements using the H3K27ac signal. Then, we calculated the fraction of the top 2,500 putative enhancers that were overlapping with feDMRs.

To test whether feDMRs can capture more enhancers than chromatin states, we computed the validation rate of the non-overlapping feDMRs. Also, we calculated the validation rate of ChromHMM enhancers by overlapping them with VISTA elements. This is used as additional baseline for evaluating feDMRs.

### Enriched TF binding motifs in tissue-specific feDMRs

To identify TF motifs that were enriched in feDMRs, we scanned the genome to delineate TF motif occurrences as previously described^33^. In brief, we used TF binding position weight matrices (PWMs) from the MEME motif database (v11, 2014 Jan 23. motif sets chen2008, hallikas2006, homeodomain, JASPAR_CORE_2014_vertebrates, jolma2010, jolma2013, macisaac_theme.v1, uniprobe_mouse, wei2010_mouse_mw, wei2010_mouse_pbm, zhao2011). Then, FIMO^64^ was used to scan the genome to identify TF motif occurrences using options ‘–output-pthresh 1E-5–max-stored-scores 500000’.

Next, we performed a hypergeometric test to identify significant motif enrichment. For each tissue type, we calculated the motif enrichment for feDMRs in that tissue (foreground) against a list of feDMRs identified for other tissues not overlapping with the foreground tissue list. For this analysis, we extended the average size of both foreground and background feDMRs to 400 bp to avoid bias due to size differences. For a given tissue *t*, the total number of foreground and background feDMRs is *N*_*f*,*t*_ and *N*_*b*,*t*_, respectively, and *N*_*t*_ = *N*_*f*,*t*_ + *N*_*b*,*t*_ is the total number of feDMRs. For a given TF binding motif *m*, TF motif occurrences are overlapped with *n*_*f*,*t*,*m*_ foreground and *n*_*b*,*t*,*m*_ background feDMRs, while *n*_*t*,*m*_ = *n*_*f*,*t*,*m*_ + *n*_*b*,*t*,*m*_ is the total number of overlapping feDMRs. The probability of observing *n*_*f*,*t*,*m*_ or more overlapping foreground feDMRs (*P*) is defined as:

$$P(X\ge {n}_{f,t,m}|{N}_{f,t},{n}_{f,t,m},{N}_{b,t},{n}_{b,t,m})=\mathop{\sum }\limits_{x={n}_{f,t,m}}^{{n}_{t,m}}\frac{(\begin{array}{c}{N}_{f,t}\\ x\end{array})(\begin{array}{c}{N}_{b,t}\\ {n}_{t,m}-x\end{array})}{(\begin{array}{c}{N}_{t}\\ {n}_{t,m}\end{array})}$$

For each tissue type, we performed this test for all motifs (*n* = 532). Then, the *P* values for each tissue were adjusted using the Benjamini–Hochberg method and the motifs were called as significant if they passed 1% false discovery rate (FDR) cutoff. Last, we excluded any TF-binding motifs whose TF expression level was less than 10 TPM. The results are listed in Supplementary Table 7.

### Enriched pathways and biological processes of feDMR neighbouring genes

For each tissue stage, we used GREAT^65^ to find enriched pathways and biological processes of genes near feDMRs identified in that tissue. For each tissue stage, GREAT was run under the ‘Single nearest gene’ association strategy on 10,000 feDMRs with the highest enhancer scores. The GREAT analysis results are listed in Supplementary Table 8.

### Enrichment of heritability in feDMRs for human diseases and traits

We applied stratified LD score regression^47^ to test for the heritability enrichment of different traits in feDMRs. The code for LD score regression was from https://github.com/bulik/ldsc (2 March 2018). LD score regression was performed on HapMap3^66^ SNPs downloaded from https://data.broadinstitute.org/alkesgroup/LDSCORE/weights_hm3_no_hla.tgz. Then, the SNP list was further filtered to the SNPs used in a pretrained baseline model (https://data.broadinstitute.org/alkesgroup/LDSCORE/1000G_Phase3_baselineLD_v1.1_ldscores.tgz). LD score was calculated using data for the European population in the 1000 Genomes project^67^ (https://data.broadinstitute.org/alkesgroup/LDSCORE/1000G_Phase3_plinkfiles.tgz) and the minor allele frequency of SNPs in this population was downloaded from https://data.broadinstitute.org/alkesgroup/LDSCORE/1000G_Phase3_frq.tgz. The summary statistics of 27 traits were downloaded from https://data.broadinstitute.org/alkesgroup/sumstats_formatted/. ‘PASS_Years_of_Education1.sumstats’ was ignored because the summary statistics of a more recent study on years of education were available.

To obtain the human orthologous regions of the CG-DMRs, we used liftOver to map mouse CG-DMRs (mm10) to hg19, requiring that at least 50% of the bases in CG-DMR could be assigned to hg19 (using option -minMatch = 0.5). In total, 1,034,801 out of 1,880,810 of mouse DMR regions (55%) could be aligned to the human genome.

Then, for each tissue sample, we overlapped the human orthologous regions of its feDMRs with SNPs in 1000 Genomes SNPs and calculated the LD score using 1000 Genomes data. However, only the LD score of SNPs in the pretrained baseline model were reported and used for later analysis. LD score was calculated using option ‘–ld-wind-cm 1’.

Last, we performed LD score regression for each trait and the feDMRs of each tissue sample with option ‘–overlap-annot’. The regression model used in the test included feDMRs and the annotations in the pretrained baseline model as before^47^. The latter was used to control for non-tissue-specific enrichment in generic regulatory elements, such as all promoters^47^. In total, we performed 1,953 tests (27 traits × 59 tissue samples). *P* values were calculated using reported coefficient *z*-score (Coefficient_*z*-score) using the R function pnorm with parameter ‘lower.tail=F’. The coefficient_*z*-score was based on 200 repeats of block jackknife resampling and thus the sample size of this statistical test is 200. To correct *P* value inflation due resulting from to multiple comparisons, we applied the Benjamini–Hochberg approach separately on the *P* values from tests on the feDMRs of each tissue sample. A *P* value cutoff given 5% FDR was used to call significant enrichment.

### Categorizing CG-DMRs

To better understand the potential functions of CG-DMRs, we grouped them into various categories on the basis of genomic location and chromatin signatures. First, we overlapped CG-DMRs with promoters, CGIs and CGI shores and defined the CG-DMRs that overlapped with these locations as proximal CG-DMRs. Out of the 153,019 proximal CG-DMRs, 46,692, 90,831, 1,710 and 13,786 overlapped with CGI promoters, non-CGI promoters, CGIs and CGI shores, respectively. We avoided assigning proximal CG-DMRs into multiple categories by prioritizing the four genomic features as CGI promoter, non-CGI promoter, CGI and CGI shores (ordered in decreasing priority). Each CG-DMR was assigned to the category with the highest priority.

We further classified the remaining 1,655,791 distal CG-DMRs as follows: (1) 397,320 of them were predicted as distal feDMRs (CG-DMRs that show enhancer-like chromatin signatures^44,68^) as described above. (2) Next, we defined flanking distal feDMRs as the CGs that were within 1 kb of distal feDMRs but were not predicted as enhancers (feDMRs). In total we found 212,620 such CG-DMRs. (3) Then, among the remaining, unclassified CG-DMRs, 159,347 CG-DMRs were identified as tissue-specific CG-DMRs in at least one of the tissues because they displayed strong tissue-specific hypomethylation patterns (mCG difference ≥ 0.3). By checking the enrichment of histone marks in their hypomethylated tissues, we found that they were enriched for H3K4me1 but not other histone marks, and these chromatin signatures resembled thsoe of primed enhancers^69^. Therefore, we defined these CG-DMRs as primed distal feDMRs. (4) Last, we defined the remaining CG-DMRs as unexplained CG-DMRs (unxDMRs) because their functional roles could yet not be assigned. We found that unxDMRs have strong overlap with transposons and we further divided them into two classes: te-unxDMRs (*n* = 449,623) and nte-unxDMRs (*n* = 436,881). te-unxDMRs are unxDMRs that overlap with transposons, and the remainder were nte-unxDMRs.

### Evolutionary conservation of CG-DMRs

The evolutionary conservation of CG-DMRs was measured using PhyloP score^70^ from the UCSC genome browser^51^ (http://hgdownload.cse.ucsc.edu/goldenpath/mm10/phyloP60way/mm10.60way.phyloP60way.bw). Next, Deeptools2^55^ was used to generate the profile of evolutionary conservation of the CG-DMR centres and ±5-kb flanking regions using options ‘reference-point–referencePoint=center -a 5000 -b 5000’.

To find the fraction of CG-DMRs that are evolutionarily conserved, we overlapped CG-DMRs from different categories with conserved DNA elements in the mouse genome. The list of conserved elements was downloaded from UCSC genome browser^51^ (phastConsElements60Way in mm10 mouse reference).

### CG-DMR effect size

We defined the effect size of a CG-DMR as the absolute difference in mCG level between the most hypomethylated tissue sample and the average of samples in the bulk. The average mCG level of some CG-DMRs in bulk samples estimates the baseline mCG level of that genomic region. The bulk samples are selected as 50% of all samples such that the range of their mCG level is narrowest (see ‘Identification of tissue-specific CG-DMRs’ for details). In this definition, the effect size indicates the degree of hypomethylation of CG-DMRs. The effect size of DMSs is defined in the same way.

### Finding TF-binding motifs enriched in flanking distal feDMRs

To identify TF-binding motifs that were enriched in flanking distal feDMRs relative to feDMRs, we performed motif analysis using the former as foreground and the latter as background. Specifically, for each tissue, the tissue-specific feDMRs were used as background, while flanking distal feDMRs that were within 1 kb of these tissue-specific feDMRs were used as foreground. To avoid potential bias resulting from differences in size distribution, both foreground and background regions were extended from both sides (5′ and 3′) such that both had a mean size of 400 bp. Next, a hypergeometric test was performed to find TF-binding motifs that were significantly enriched in the foreground. This test was the same as that used for the identification of TF-binding motifs in feDMRs.

### TF-binding motif enrichment analysis for primed distal feDMRs

We also performed motif analysis to identify TF-binding motifs that were enriched in primed distal feDMRs. The procedure was similar to the motif enrichment analysis on feDMRs. For each tissue, the primed distal feDMRs that were hypomethylated in that tissue were considered as foreground while the remaining primed distal feDMRs were considered as background. Then, a hypergeometric test was performed to identify significant motif enrichment.

Next, for each tissue type, we compared the TF-binding motifs that were enriched in primed distal feDMRs and the tissue-specific feDMRs. The hypergeometric test was used to test the significance of overlap—the chance of obtaining the observed overlap if the two lists were based on random sampling (without replacement) from the TF-binding motifs with TF expression level greater than 10 TPM.

### Monte Carlo test of the overlap between unxDMRs and transposons

To estimate the significance of overlap between unxDMRs and transposable elements (TEs), we shuffled the location of unxDMRs using the ‘shuffleBed’ tool from bedtools^61^ with default setting and recalculated the overlaps. After repeating this step 1,000 times, we obtained an empirical estimate of the overlap if unxDMRs were randomly distributed in the genome. Let the observed number of TE-overlapping unxDMRs be *x*^obs^ and the number of TE-overlapping shuffled unxDMRs in permutation *i* be ${x}_{i}^{{\rm{permut}}}$. We then calculated *P* values as

$$P=\frac{[{\sum }_{i=1}^{1,000}I({x}^{{\rm{obs}}}\le {x}_{i}^{{\rm{permut}}})]+1}{1,000+1}$$

in which $$I(x)=\{\begin{array}{c}1\\ 0\end{array}\,\begin{array}{c}x={\rm{true}}\,\\ x={\rm{false}}\end{array}$$.

### Identification of large hypo CG-DMRs

Large hypo CG-DMRs were called using the same procedure as previously described^33^. For each tissue type, tissue-specific CG-DMRs were merged if they were within 1 kb of each other. Then, we filtered out merged CG-DMRs less than 2 kb in length.

We overlapped genes with large hypo CG-DMRs and then filtered out any genes with names starting with ‘Rik’ or ‘Gm[0-9]’, in which [0-9] represents a single digit, because the ontology of these genes was ill-defined.

### Super-enhancer calling

Super-enhancers were identified using the ROSE^36,71^ pipeline. First, H3K27ac peaks were called using MACS2^56^ callpeak module with options ‘–extsize 300 -q 0.05–nomodel -g mm’. Control data were used in the peak-calling step. Next, ROSE was run with options ‘-s 12500 -t 2500’, and H3K27ac peaks, mapped H3K27ac ChIP–seq reads and mapped control reads as input. The super-enhancer calls were generated for each tissue sample. Then, we obtained the super-enhancers for one tissue type by merging the super-enhancers called at each stage of fetal development (E10.5 to P0). Last, we generated a list of merged super-enhancers by merging super-enhancer calls for all tissue types except liver.

### Quantification of mCG dynamics in tissue-specific CG-DMRs

To quantify mCG dynamics, we defined and counted loss-of-mCG and gain-of-mCG events. A loss-of-mCG or gain-of-mCG event is a decrease or increase, respectively, in mCG level by at least 0.1 in one CG-DMR in one stage interval. For example, if the mCG levels of one CG-DMR at E11.5 and E12.5 are 0.8 and 0.7, respectively, in heart, it is considered a loss-of-mCG event at stage interval E11.5–E12.5. A stage interval is defined as the transition between two sampled adjacent stages (for example, E15.5 and E16.5).

### Clustering forebrain-specific CG-DMRs based on mCG and H3K27ac dynamics

We used *k*-means clustering to identify subgroups of forebrain-specific CG-DMRs on the basis of mCG and H3K27ac dynamics. First, for each forebrain-specific CG-DMR, we calculated the mCG level and H3K27ac enrichment in forebrain samples from E10.5 to adult stages. Here, we used published methylome data for postnatal 1-, 2- and 6-week frontal cortex^9^ to approximate the DNA methylation landscape of the adult forebrain. We also incorporated H3K27ac data for postnatal 1-, 3- and 7-week forebrain samples. Next, to make the range of H3K27ac enrichment values comparable to that of mCH levels, for each forebrain-specific CG-DMR, the negative H3K27ac enrichment values were thresholded as zero and then each value was divided by the maximum. If the maximum was zero for some forebrain-specific CG-DMRs, we set all values to be zero. *k*-means clustering of subgroups was carried out but no new patterns were observed. Last, we used GREAT^65^ employing the ‘Single nearest gene’ association strategy to identify the enriched gene ontology terms of genes near CG-DMRs for each subgroup.

### Association between mCG level and H3K27ac enrichment

To investigate the association between mCG and H3K27ac, for each tissue and each developmental stage, we first divided the tissue-specific CG-DMRs into three categories on the basis of mCG methylation levels: H (high CG methylation; mCG level > 0.6), M (moderate CG methylation; 0.2 < mCG level ≤ 0.6) and L (low CG methylation; mCG level ≤ 0.2). Then, we examined the distribution of H3K27ac enrichment in different groups of CG-DMRs by counting the number of CG-DMRs for each of four levels of H3K27ac: [0,2], (2, 4], (4, 6] and (6, ∞).

### DMV identification

We identified DMVs as previously described^37^. First, the genome was divided into 1-kb non-overlapping bins. Then, for each tissue sample (replicate), consecutive bins with an mCG level of less than 0.15 were merged into blocks; bins with no data (no CG sites or no reads) were skipped. Next, any blocks merged from at least five with-data bins were called as DMVs. For each tissue sample, we filtered for DMVs that were reproducible in two replicates by first selecting the DMVs identified in one replicate that overlapped any DMVs called in the other replicate, and then merging overlapping DMVs. Using this strategy, we obtained DMV calls for each tissue from each developmental stage. Last, we generated a list of merged DMVs for all tissue samples by merging all DMVs identified in any tissues from any developmental stages.

We overlapped genes with DMVs and then filtered out any genes with names starting with ‘Rik’ or ‘Gm[0–9]’, where [0–9] represents a single digit, because the ontology of these genes was ill-defined.

### PMD identification

PMDs were identified as previously described^8^ using a random forest classifier. To train the classifier, we first visually selected regions on chromosome 19 as strong candidates for PMDs or non-PMDs in E14.5 liver samples. Specifically, we manually annotated five PMDs that showed an obviously lower mCG level compared to adjacent genomic regions (chr19: 46110000–46240000, chr19: 45820000–45960000, chr19: 47140000–47340000 and chr19: 48060000–52910000) and seven non-PMD regions (chr19: 4713800–4928700, chr19: 7420700–7541100, chr19: 8738100–8967000, chr19: 18633300–18713800, chr19: 53315500–53390000, chr19: 55256600–55633900 and chr19: 59281600–59329200).

Next, these regions were divided into 10-kb non-overlapping bins and we calculated the percentiles of the methylation levels at the CG sites within each bin. CG sites that were within CGIs, DMVs^37^ or any of four Hox loci (see below) were excluded as these regions are typically hypomethylated which may result in incorrect PMD calling. Additionally, sites with fewer than five reads covered were also excluded. We trained the random forest classifier using data from E14.5 liver (combining the two replicates) and we then predicted whether a 10-kb bin was a PMD or non-PMD in all liver samples (considering replicates separately). We chose a large bin size (10 kb) to reduce the effect of smaller-scale variations in methylation (such as DMRs) as PMDs were first discovered as large (mean length 153 kb) regions with intermediate methylation level (<70%)^7^. Furthermore, the features (the distribution of methylation level of CG sites, which measured the fraction of CG sites that showed methylation levels at various methylation level ranges) used in the classifier required enough CG sites within each bin to robustly estimate the distribution, which necessitated a relatively large bin. Also, we excluded any 10-kb bins containing fewer than ten CG sites for the same reason. These percentiles were used as features for the random forest. The random forest implement was from scikit-learn (version 0.17.1)^72^ python module and the following arguments were supplied to the Python function RandomForestClassifier from scikit-learn: n_estimators = 10000, max_features = None, oob_score = True, compute_importances = True.

Last, we merged consecutive 10-kb bins that were predicted as PMDs into blocks and filtered out blocks smaller than 100 kb. We further excluded blocks that overlapped with gaps in the mm10 genome (downloaded from UCSC genome browser, 21 September 2013). To obtain a set of PMDs that was reproducible in both replicates, we considered only genomic regions that were larger than 100 kb and were covered by PMD calls in both replicates. These regions were the final set of PMDs used for later analyses. Because there was only one replicate for adult liver, we called the PMDs at this stage using the single replicate.

PMDs were originally called using the above procedure without excluding CG sites in Hox gene clusters. However, because these Hox loci are more likely to be considered as large DMVs^37^, we removed any PMDs that overlapped with the four Hox clusters (chr11: 96257739–96358516, chr15: 102896908–103038064, chr2: 74648392–74748841 and chr6: 52146273–52277140).

### Overlap between PMDs and lamina-associated domains (LADs)

To examine the relationship between PMDs and LADs in normal mouse liver cells (AML12 hepatocytes) we used LAD data from supplementary table 2 of ref. ^73^. The mm9 coordinates of LADs were converted to mm10 using liftOver with default settings. We then used Monte Carlo testing to examine the significance of the overlap between PMDs and LADs. Similar to the procedure for checking the overlap between TEs and unxDMRs, we permutated (1,000 times) the genomic locations of PMDs and recorded the number of overlapping bases (${x}_{i}^{{\rm{shuf}}}$for permutation *i*) between shuffled PMDs and LADs. Then, we compared ${x}_{i}^{{\rm{shuf}}}$ with the observed numbers of overlapping bases (*x*^obs^) between PMDs and LADs and computed *P* values as:

$$P=\frac{[{\sum }_{i=1}^{1000}I({x}^{{\rm{obs}}}\le {x}_{i}^{{\rm{shuf}}})]+1}{1,000+1}$$

in which $$I(x)=\{\begin{array}{c}1\\ 0\end{array}\begin{array}{c}x={\rm{true}}\\ x={\rm{false}}\end{array}$$.

### Replication timing data

Replication timing data (build mm10) for three mouse cell types was used from ReplicationDomain^74^. The cell types used for these analyses were mES cells (id: 1967902&4177902_TT2ESMockCGHRT), neural progenitor cells (id: 4180202&4181802_TT2NSMockCGHRT) and mouse embryonic fibroblasts (id: 304067-1 Tc1A).

### Gene expression in PMDs

We obtained information about PMD-overlapping protein-coding genes using ‘intersectBed’. A similar approach was used to identify protein-coding genes that overlapped with PMD flanking regions (100 kb upstream and downstream of PMDs); genes that overlapped with PMDs were removed from this list. Last, we compared the expression of PMD-overlapping genes (*n* = 5,748) and genes (*n* = 2,555) that overlapped flanking regions.

### Sequence context preference of mCH

To interrogate the sequence preference of mCH, as previous described^8^, we first identified CH sites that showed a significantly higher methylation level than the low level noise (which was around 0.005 in term of methylation level) caused by incomplete sodium bisulfite non-conversion. For each CH site, we counted the number of reads that supported methylation and the number of reads that did not. Next, we performed a binomial test with the success probability equal to the sodium bisulfite non-conversion rate. The FDR (1%) was controlled using the Benjamini–Hochberg approach^75^. This analysis was independently performed for each three-nucleotide context (for example, a *P* value cutoff was calculated for CAG cytosines). Last, we counted sequence motif occurrence of ± 5bp around the trinucleotide context of methylated mCH sites and visualized the sequence preferences using seqLogo^76^.

### Calling mCH domains

We used an iterative process to call mCH domains, which are genomic regions that are enriched for mCH compared to flanking regions. First, we selected a set of samples that showed no evidence of mCH. Data from these samples were used in the following steps to filter out genomic regions that are prone to misalignment and showed suspicious mCH abundance. Analysis of the global mCH level and mCH motifs revealed that E10.5 and E11.5 tissues (excluding heart samples) have extremely low mCH and the significantly methylated non-CG sites showed little CA preference. Therefore, we assumed that these sites contain no mCH domain and any mCH domains called in control samples by the algorithm were likely to be artefacts. By filtering out the domains called in the control samples, we were able to exclude the genomic regions that were prone to mapping error and avoid other potential drawbacks in the processing pipeline.

To identify genomic regions in which sharp changes in mCH levels occurred, we applied a change point detection algorithm with the mCH levels of all 5-kb non-overlapping bins across the genome as input. We included only bins that contained at least 500 CH sites and in which at least 50% of CH sites were covered by 10 or more reads. The identified regions defined the boundaries that separate mCH domains from genomic regions that show background mCH levels. We implemented this step using the function cpt.mean in R package ‘changepoint’, with options ‘method=”PELT”, pen.value = 0.05, penalty = ”Asymptotic” and minseglen = 2’. To match the range of chosen penalty, we scaled up mCH levels by a factor of 1,000.

The iterative procedure was carried out as follows: 1) An empty list of excluded regions was created. 2) For each control sample, the change point detection algorithm was applied to the scaled mCH levels of 5-kb non-overlapping bins. Bins that overlapped excluded regions were ignored. 3) The genome was segmented into chunks based on identified change points. 4) The mCH level of each chunk was calculated as the mean mCH level of the overlapping 5-kb bins that did not overlapped excluded regions. 5) mCH domains were identified as chunks whose mCH level was at least 50% greater than the mCH level of both upstream and downstream chunks. A pseudo-mCH level of 0.001 was used to avoid dividing by zero. 6) mCH domains were added to the list of excluded regions. 7) Steps 2 to 6 were repeated until the list of excluded regions stopped expanding. 8) Steps 2 to 5 were then applied to all samples. 9) For each tissue or organ, only regions were retained that were identified as (part of) an mCH domain in both replicates, and regions less than 15 kb in length were filtered out; mCH domains must span at least three bins. The above criterion were used to define mCH domains for each tissue or organ. 10) Individual mCH domains from each tissue and organ were merged to obtain a single combined list of 384 mCH domains.

### Clustering of mCH domains

We applied *k*-means clustering to group the 384 identified mCH domains into 5 clusters on the basis of the normalized mCH accumulation profile of each mCH domain and corresponding flanking regions (100 kb upstream and 100 kb downstream). Specifically, 1) in each tissue sample, the mCH accumulation profile of one mCH domain was represented as a vector of length 50: the mCH levels of 20 5-kb bins upstream of the mCH domain, 10 bins that equally divided the mCH domain and 20 5-kb bins downstream. 2) Then, we normalized all values by the average mCH levels of bins of flanking regions (the 20 5-kb bins upstream and 20 5-kb bins downstream of the mCH domain). 3) We next computed the profile in samples of the six tissue types (midbrain, hindbrain, heart, intestine, stomach and kidney) that showed the most evident mCH accumulation in fetal development. 4) Using the profile of these tissue samples, *k*-means (R v3.3.1) was used to cluster mCH domains with *k* = 5. We also tried higher cluster numbers (for example, 6) but did not identify any new patterns. Even using the current *k* setting (*k* = 5), the mCH domains in clusters 1 (C1) and 3 (C3) shared a similar mCH accumulation pattern.

### Genes in mCH domains

We obtained the overlapping gene information for each of the mCH domains by overlapping gene bodies with mCH domains using ‘intersectBed’ in bedtools^61^. Only protein-coding genes were considered. We further filtered out any genes with names starting with ‘Rik’ or ‘Gm[0–9]’, where [0–9] represents a single digit, because the ontology of these genes was ill-defined. For the overlapping genes of each mCH domain cluster, we used EnrichR^49,77^ to find the enriched gene ontology terms (‘GO_Biological_Process_2015’).

Next we asked whether the identified overlapping genes were enriched for TF-encoding genes. For this purpose, a list of mouse TFs from AnimalTFDB^78^ (27 February 2017) was used. We then performed a Monte Carlo test to estimate the significance of the findings. Specifically, *x*^obs^ is the number of TF-encoding genes in all overlapping genes. We randomly selected (1,000 times) the same number of genes and, on the *i*th time, ${x}_{i}^{{\rm{permut}}}$ of the randomly selected genes encoding TFs. Last, the *P* value was calculated as

$$P=\frac{[{\sum }_{i=1}^{1,000}I({x}^{{\rm{obs}}}\le {x}_{i}^{{\rm{permut}}})]+1}{1,000+1}$$

in which $$I(x)=\{\begin{array}{c}1\\ 0\end{array}\,\begin{array}{c}x={\rm{true}}\,\\ x={\rm{false}}\end{array}$$.

### mCH accumulation indicates gene repression

To evaluate the association between mCH abundance and gene expression, we traced the expression dynamics of genes inside mCH domains. For mCH domains in each cluster, we first calculated the TPM *z*-score for each of the overlapping genes. Specifically, for each tissue type and each overlapping gene, we normalized TPM values in the samples of that tissue type to *z*-scores. The *z*-scores showed the trajectory of dynamic expression, in which the aptitude information of expression was removed. If the gene was not expressed, we did not perform the normalization. Next, we calculated the *z*-scores for all genes that had no overlap with any mCH domain. Last, we subtracted the *z*-scores of overlapping genes from the *z*-scores of all genes outside mCH domains. The resulting values indicated the level of expression of genes in mCH domains relative to genes not in mCH domains.

### Weighted correlation network analysis

We used WGCNA^79^, an unsupervised method, to detect sets of genes with similar expression profiles across samples (R package, ‘WGCNA’ version 1.51). In brief, TPM values were first log_2_ transformed (with pseudo count 1 × 10^−5^). Then, the TPM value of every gene across all samples was compared against the expression profile of all other genes and a correlation matrix was obtained. To obtain connection strengths between any two genes, we transformed this matrix to an adjacency matrix using a power adjacency function. To choose the parameter (soft threshold) of the power adjacency function, we used the scale-free topology (SFT) criterion, where the constructed network is required to at least approximate scale-free topology. The SFT criterion recommends use of the first threshold parameter value at which model-fit saturation is reached as long as it is above 0.8. In this study, the threshold was reached for a power of 5.

Next, the adjacency matrix is further transformed to a topological overlap matrix (TOM) that finds ‘neighborhoods’ of every gene iteratively, based on the connection strengths. The TOM was calculated on the basis of the adjacency matrix derived using the signed hybrid network type, biweight mid correlation and signed TOMtype parameters of the TOMsimilarityFromExpr module in WGCNA. Hierarchical clustering of the TOM was done using the flashClust module using the average method. Next, we used the cutreeDynamic module with the hybrid method, deepSplit = 3 and minClusterSize = 30 parameters to identify modules that have at least 30 genes. A summarized module-specific expression profile was created using the expression of genes within the given module, represented by the eigengene. The eigengene is defined as the first principal component of the log_2_ transformed TPM values of all genes in a module. In other words, this is a virtual gene that represents the expression profile of all genes in a given module. Next, very similar modules were merged after a hierarchical clustering of the eigengenes of all modules with a distance threshold of 0.15. Finally, the eigengenes were recalculated for all modules after merging.

### Gene ontology analysis of genes in CEMs

To better understand the biological processes of genes in each CEM, we used Enrichr^49,77^ (http://amp.pharm.mssm.edu/Enrichr/) to identify the enriched gene ontology terms in the GO_Biological_Process_2015 category.

### Correlating eigengene expression with mCG and enhancer scores of feDMRs

We investigated the association between gene expression and epigenomic signatures of regulatory elements in CEMs. First, for each CEM, we used the eigengene expression to summarize the transcription patterns of all genes in the module. Then, we calculated the normalized average enhancer score and normalized average mCG level of all feDMRs that were linked to the genes in the CEM. Specifically, to reduce the potential batch effect, for each tissue and each stage, we normalized the enhancer score of each feDMR by the mean enhancer score of all feDMRs. mCG levels of feDMRs were normalized in similar way except that the data of all DMRs was used to calculate the mean mCG level for each tissue and each stage. Next, for each CEM, the TPM of its eigengene, the normalized average enhancer score and the mCG level of linked feDMRs were converted to *z*-scores across all fetal stages for each tissue type (for analysis of tissue-specific expression) or across tissue types for each development stage (for analysis of temporal expression). Last, for each CEM, we calculated the Pearson correlation coefficient (R 3.3.1) between the *z*-score of eigengene expression and the *z*-score of normalized enhancer score (or mCG level) for each module. The correlation coefficients were calculated for two different settings: 1) for each tissue type, the correlation was computed using the *z*-score of normalized eigengene expression values and enhancer scores (or mCG levels) across different development stages; or 2) for each developmental stage, the correlation was computed across different tissue types. The coefficients from the former analysis indicate how well temporal gene expression is correlated with enhancer score or mCG level of regulatory elements, while the latter measures the association with tissue-specific gene expression.

We then tested whether the correlation that we observed was significant by comparing it with the correlation based on shuffled data. In the analysis of tissue-specific expression in a given tissue type, we mapped the eigengene expression of one CEM to the enhancer score (or mCG level) of feDMRs linked to genes in a randomly chosen CEM. For example, in the shuffle setting, when the given tissue type was heart, we calculated the correlation between the eigengene expression of CEM14 and the enhancer score of the feDMRs linked to genes in CEM6. In the analysis of temporal expression, given a specific developmental stage, we performed a similar permutation. Next, we calculated the Pearson correlation coefficients for this permutation setting. Last, using a two-tailed Mann–Whitney test, we compared the median of observed correlation coefficients and the median of those based on shuffled data.

### Reporting summary

Further information on research design is available in the Nature Research Reporting Summary linked to this paper.

## Online content

Any methods, additional references, Nature Research reporting summaries, source data, extended data, supplementary information, acknowledgements, peer review information; details of author contributions and competing interests; and statements of data and code availability are available at 10.1038/s41586-020-2119-x.

## Supplementary information

Supplementary Information This file contains 4 Supplementary Notes, which contain additional details to the text and data presented in the main text.

Reporting Summary

Supplementary Information This file contains the detailed tissue excision procedure for all embryonic and postnatal mouse tissues surveyed in this study. It includes the protocols of experiment preparation, embryo collection, embryo stage assessing and tissue excision/preparation. This document also includes the specific anatomical definition of tissues on every developmental stage.

Supplementary Information This file describes the detailed protocols of tissue fixation, sonication and DNA extraction, including several steps: pulverization of tissue, cross-linking of tissues, sonication of tissues and DNA isolation (precipitation and chromatin fragmentation check).

Supplementary Data This is a compressed file containing the feDMRs of every non-liver tissue on every developmental stage. The feDMRs of each tissue sample are stored in a single file. After decompression, see “README.txt” file for details of file format.

Supplementary Table This file contains Supplementary Table 1.

Supplementary Table This file contains Supplementary Table 2.

Supplementary Table This file contains Supplementary Table 3.

Supplementary Table This file contains Supplementary Table 4.

Supplementary Table This file contains Supplementary Table 5.

Supplementary Table This file contains Supplementary Table 6.

Supplementary Table This file contains Supplementary Table 7.

Supplementary Table This file contains Supplementary Table 8.

Supplementary Table This file contains Supplementary Table 9.

Supplementary Table This file contains Supplementary Table 10.

Supplementary Table This file contains Supplementary Table 11.

Supplementary Table This file contains Supplementary Table 12.

## Data Availability

All WGBS data from mouse embryonic tissues are available at the ENCODE portal (https://www.encodeproject.org/) and/or have been deposited in the NCBI Gene Expression Omnibus (GEO; Supplementary Table 1). The additional RNA-seq data for forebrain, midbrain, hindbrain and liver are available at the GEO under accession GSE100685. All other data used in this study, including ChIP–seq), ATAC–seq, RNA-seq and additional WGBS data, are available at the ENCODE portal and/or GEO (Supplementary Table 2).

## Code availability

methylpy (1.0.2) and REPTILE (1.0) are available at https://github.com/yupenghe/methylpy and https://github.com/yupenghe/REPTILE, respectively. Custom code used for this study is available at https://github.com/yupenghe/encode_dna_dynamics. This work used computation resources from the Extreme Science and Engineering Discovery Environment (XSEDE)^80^.
