# Supplementary material for: Spatiotemporal DNA methylome dynamics of the developing mouse fetus
Source: Nature. 2020 Jul 29;583(7818):752–9. doi: 10.1038/s41586-020-2119-x (PMC7398276; doi:10.1038/s41586-020-2119-x)
Supplement: Supplementary file 1 — This file contains 4 Supplementary Notes, which contain additional details to the text and data presented in the main text. [file 41586_2020_2119_MOESM1_ESM.pdf]

nature

https://doi.org/10.1038/s41586-020-2119-x

Supplementary information

Spatiotemporal DNA methylome dynamics of the developing mouse fetus

In the format provided by the authors and unedited

Yupeng He, Manoj Hariharan, David U. Gorkin, Diane E. Dickel, Chongyuan Luo, Rosa G. Castanon, Joseph R. Nery, Ah Young Lee, Yuan Zhao, Hui Huang, Brian A. Williams, Diane Trout, Henry Amrhein, Rongxin Fang, Huaming Chen, Bin Li, Axel Visel, Len A. Pennacchio, Bing Ren & Joseph R. Ecker✉

Table of Contents

Supplementary Notes **1**
Supplementary Note 1 - mCG landscape remodeling in fetal liver *1*
Supplementary Note 2 - Annotating methylation variable regions *2*
Supplementary Note 3 - CG-DMR effect size *4*
Supplementary Note 4 - Evaluation of fetal enhancer-like DMRs *5*
References **7**

Supplementary Notes

Supplementary Note 1 - mCG landscape remodeling in fetal liver

Distinct from the hypermethylated genome of all other tissues, the liver genome underwent drastic global demethylation from E11.5 to E14.5, and remained hypomethylated until E16.5, after which it returned to a hypermethylated state at P0 (Fig. 1b). The hypomethylated liver genome, present during E12.5 to E16.5, displayed a partially methylated domains (PMDs) signature, a methylation feature previously observed in human cultured cell lines[7,79], pancreas[8], placenta[80] and cancer samples[81,82]. A recent study[83] also reported hypomethylation in E18.5 mouse liver. PMDs are large genomic regions (typically greater than 100kb) that are lowly CG methylated (Extended Data Fig. 1a). PMDs displayed stronger hypomethylation than flanking regions (Extended Data Fig. 1b). We systematically identified PMDs in liver samples from all stages (Methods). Strikingly, from E14.5 to E16.5, PMDs covered more than half of the genome and the coverage shrunk dramatically afterwards (Extended Data Fig. 1b). We found that PMDs identified in E15.5 displayed hypomethylation in all liver samples and covered almost all PMDs from other stages (Extended Data Fig. 1c-d). These results indicate that the PMDs identified at different fetal stages are essentially identical and the different PMDs calls were due to various signal-to-noise ratios. Therefore, we defined the PMDs identified in E15.5 liver as liver PMDs (n = 4,578; average size = 338kb).

Mouse liver PMDs share all molecular signatures of the PMDs identified in human fibroblast cell lines, normal and cancer tissues[7,8,81,82]: First, mouse PMDs are enriched for H3K9me3 and H3K27me3 and are depleted of H3K27ac (Extended Data Fig. 1e). Second, mouse PMDs tend to be replicated during the later stages of the cell cycle and strongly overlap with lamina-associated domains (Extended Data Fig. 1f; p-value < 0.001; Monte Carlo testing; Methods). Furthermore, we found that genes overlapping with mouse PMDs tend to have lower

expression compared to genes outside PMDs, which was also reported by Schultz et al. for human pancreas[8]. These shared properties indicate that human and mouse PMDs are likely identical genome feature and their presence may be due to similar mechanism, likely the failure of mCG maintenance in rapidly dividing cells[81].

The presence of PMDs in fetal liver coincides with hematopoiesis[84,85]. Hematopoiesis initiates at E11.5, while the liver genome remains hypermethylated (Fig. 1b). Then, hematopoietic expansion occurs between E12.5 and E14.5, during which the liver genome underwent demethylation and PMDs became evident (Fig. 1b; Extended Data Fig. 1b,d). The increasing number of rapidly dividing cells during this expansion period may explain the formation of PMDs. After E15.5, hematopoiesis declines although the "liver tissue" genome is not fully remethylated until P0 (Extended Data Fig. 1b).

Supplementary Note 2 - Annotating methylation variable regions

We categorized, in total, 1,808,810 fetal CG-DMRs based on their chromatin states, mCG effect size and their relationship with other genomic features. We found that 8.5% (n = 153,019 of CG-DMRs overlapped with known promoters, CpG islands (CGIs) or CGI shores. We termed these CG-DMRs as proximal CG-DMRs (Fig. 1d; Extended Data Fig. 2c-f). The remaining vast majority of CG-DMRs (~91.5% or 1,655,791) were distally located and showed a high degree of evolutionary conservation, suggesting that they are functional (Fig. 1d; Extended Data Fig. 2e-g). We used the REPTILE[40] method to predict fetal enhancer-linked CG-DMRs (feDMRs), which are CG-DMRs that showed enhancer-like chromatin signatures (Fig. 4a). 85% (397,320) of them are distal to known promoters and we referred them as distal feDMRs (Fig. 1d).

In addition to distal feDMRs, we also identified 212,620 CG-DMRs that flank (within 1kb) distal feDMRs but which are not themselves predicted as enhancers (Fig. 1d). These flanking distal feDMRs (fd-feDMRs) are much less conserved than feDMRs although their mCG level is moderately correlated with nearby feDMRs (median Pearson correlation coefficient = 0.46, n = 212,620; Extended Data Fig. 2e-g; see Methods), suggesting that a fraction of fd-feDMRs may be the by-product of demethylation of the adjacent feDMRs. Alternatively, fd-feDMRs may be bound by pioneer TF(s) which allow opening of chromatin of adjacent

feDMRs[86,87] . This notion is supported by the enrichment of binding motifs for several known pioneer TFs[86] such as FOXA2 and GATA3 at fd-DMRs (Supplementary Table 11). Interestingly, binding motifs of the insulator protein CTCF and several transcriptional repressors (e.g. CUX1[88]) are also enriched at fd-feDMRs, indicating a third possibility that fd-feDMRs correspond to insulator and silencer elements (Supplementary Table 11).

Besides the above CG-DMR classes, another type of distal CG-DMR (n = 159,347) are the primed distal feDMRs (pd-feDMRs); these display strong CG hypomethylation in at least one tissue sample and are linked to primed fetal enhancers[68] (mCG difference ≥ 0.3; Fig. 1d; Extended Data Fig. 3a; Methods). In the tissues where they are hypomethylated, pd-feDMRs showed chromatin signatures resembling primed enhancers[68] which are enriched for H3K4me1 while lacking H3K27ac and H3K27me3 (Extended Data Fig. 3b). Like feDMRs, pd-feDMRs are also evolutionary conserved (Extended Data Fig. 2e-g). Consistent with their putative role as enhancers, and similar to feDMRs, they share significant TF-binding motif signature enrichment in 9 out of the 12 tissue types (Extended Data Fig. 3c; Supplementary Table 12).

The remaining unclassified distal CG-DMRs (886,504) show more subtle CG hypomethylation patterns, suggesting that they likely derive from a small fraction of cells within these complex tissues/organs (Extended Data Fig. 3d-e; Methods). Since a functional role cannot yet be assigned, we named this group “unexplained CG-DMRs” (unxDMR). The genomic locations of unxDMRs significantly overlapped with transposons (50.7%, fold-enrichment = 1.46, p-value < 0.001, Monte Carlo testing; Methods). Inspired by this observation, we divided unxDMRs into two subgroups: unxDMRs that overlapped with TE (te-unxDMRs) and ones not overlapping (nte-unxDMRs) (Extended Data Fig. 3d-e). te-unxDMRs were less evolutionarily conserved compared to flanking regions but transposons may be a source of novel regulatory elements[89]. Different from te-unxDMRs, genomic sequences underling nte-unxDMRs are as conserved as feDMRs, implying that they may be functional. Indeed, 10% and 45% of the nte-unxDMRs showed open chromatin in purified neurons[30] and a variety of mouse cell lines and tissues[90], respectively (fold-enrichment = 1.10 and 1.16, p-value < 0.001, Monte Carlo testing; Methods). Therefore, nte-unxDMRs are likely regulatory elements active only in rare cell types and their weak hypomethylation profiles are due to the tissue heterogeneity, which are supported by previous studies of cell-specific[30] and single-cell methylomes[17]. It is also possible that mCG in nte-unxDMRs underwent dynamic regulation in a large portion of cells and only the average

effect is measured. Nonetheless, single-cell level profiling will be essential for uncovering the source of these enrichments.

Supplementary Note 3 - CG-DMR effect size

Although all CG-DMRs displayed statistically significant mCG variation, the degree of mCG difference (effect size) varies. Considering mCG is pervasive in mammalian genome and CG hypomethylation is indicative of regulatory activity, we defined the effect size of a CG-DMR as the absolute difference between the lowest mCG level and the average mCG level of "bulk samples" (selected half of the samples with smallest mCG level range; See Methods for details). 71% of the CG-DMRs have effect size greater than 0.2 (Extended Data Fig. 4a), indicating that at least 20% of cells from the most hypomethylated tissue sample contain unmethylated allele. The mean effect size of CG-DMRs is 0.284, and 37% of the CG-DMRs passed the widely used 0.3 effect size cutoff[10] (Extended Data Fig. 4a).

The effect size distribution varies in CG-DMRs from different categories. Primed distal feDMRs showed generally larger effect size as they were selected for strong tissue-specific CG hypomethylation (Extended Data Fig. 4b). The unexplained CG-DMRs were selected in the opposite way and displayed smaller effect size.

Each CG-DMR contains multiple CG sites, and we found most of them show differential methylation. On average, there are 9 differentially methylated sites (DMSs) in one CG-DMRs (Extended Data Fig. 4c). In 62% of CG-DMRs, more than 80% of CG sites are differentially methylated (Extended Data Fig. 4d). Furthermore, in 57% of CG-DMRs, DMSs occupy at least 80% of CG sites even if we require the effect size to be greater than or equal to 0.2 (Extended Data Fig. 4d). The CG-DMRs with more DMSs likely show stronger regulatory activity (stronger enrichment of H3K4me1 and H3K27ac, higher enhancer score, and higher transcript abundance of nearest gene) (Extended Data Fig. 4e).

CG-DMRs with larger effect size are present in more cells in the heterogeneous tissue samples, and are less likely a result of noise. Therefore, as expected, CG-DMRs that show larger effect size and contain more DMSs tend to display stronger anti-correlation with active chromatin marks, enhancer score and the transcription of nearby genes (Extended Data Fig. 4f; Methods).

Supplementary Note 4 - Evaluation of fetal enhancer-like DMRs

To validate fetal enhancer-like DMRs (feDMRs), we overlapped feDMRs with experimentally validated DNA elements from VISTA enhancer browser[25] (VISTA elements). However, the VISTA elements were biasedly selected. Compared to randomly selected sequences, they are more enriched for enhancers, which will lead to an overestimation of the true positive rate. The true positive rate for a given set of enhancer predictions is defined as the fraction of the VISTA elements that are experimentally validated enhancers (positives) among all VISTA elements that overlap with any enhancer prediction. For example, if 60% of VISTA elements are enhancers and we randomly select a subset of them, 60% of the selected elements are expected to be true positives. Therefore, the true positive rate of a set of enhancer predictions is inflated if we validate the enhancer predictions using a dataset with an inflated fraction of elements being positives.

To reduce the impact of selection bias, we need to first estimate the fraction of VISTA elements that are positives (positive rate) in a given tissue if there is minimal selection bias. We termed this fraction as genuine positive rate. Then, we can sample the current VISTA dataset to construct datasets with positive rate matching the genuine positive rate. Since the positive rate is not inflated in the constructed datasets, it will allow a fair evaluation of our enhancer prediction approach.

There are two sources of selection bias: 1) VISTA elements are selected to be enhancers of a given tissue or 2) to be enhancers of biologically related tissue(s). The second source increased the positive rate of the given tissue because enhancers of similar tissues are often shared. For example, selected forebrain enhancers will likely be also active in midbrain. Both sources will inflate the positive rate of the given tissue to be higher than if VISTA elements are randomly selected.

To begin with, we sought to identify a tissue, in which the enhancer activity of VISTA elements is conditionally independent of their enhancer activity in other tissues. Specifically, let N be the total number of VISTA elements and $a_{i,t}$ be the enhancer activity of element i in E11.5 tissue t. $a_{i,t}=0$ means element i is inactive in tissue t, whereas $a_{i,t}=1$ indicates element I is active and is able to drive reporter gene expression in tissue t. Therefore, the positive rate of tissue t is

$\sum_{i=1}^{N} a_{i,t}$. We tried to identify a tissue *t* where $\sum_{i \in C_d} a_{i,\square}$ is relatively constant, where d is a tissue different from *t* and $C_d = \{j | a_{j,d} = 1\}$. Such conditional independence ensures that selecting enhancers for other tissues has little impact of the positive rate of this tissue, i.e. the tissue is resistant to the second source of selection bias. Given that the first source of selection still exists, the positive rate of that tissue is expected to be higher than the genuine positive rate and is the upper bound of the genuine positive rate. To interrogate the conditional independence of positive rate in two different tissues, we calculated the positive rates in one tissue for the VISTA elements that are active (or inactive) in the other tissue (Extended Data Fig. 9b). As expected, the positive rate of VISTA elements in a given tissue is dependent on their enhancer activity in a biologically related tissue. For example, a higher fraction of VISTA elements are forebrain enhancers if they are active in midbrain compared to if they are inactive in midbrain. In contrast, VISTA elements active in heart are less likely to be active in brain tissues. Interestingly, we found that the positive rate of VISTA elements in limb have little dependency on their enhancer activity in other tissues. The positive rate in limb is 10% and we used this number as the upper bound of the genuine positive rate (Extended Data Fig. 9b-c).

Next, we sought to determine the lower bound of the genuine positive rate. The idea is that the genuine positive rate is equal to or higher than the positive rate of genomic regions that show little evidence of enhancers, which we termed "random positive rate". To estimate random positive rate, we started with all VISTA elements and removed the ones showing enhancer-like chromatin state and/or high degree of evolutionary conservation. Specifically, we first filtered out the VISTA elements that overlap with any feDMRs or any H3K27ac peaks. Then, from the remaining VISTA elements, we removed the ones that are among top 10% VISTA elements ranked in descending order by their PhyloP score, a type of evolutionary conservation score[69]. Lastly, we calculated the positive rate of the filtered set of VISTA elements, which is the random positive rate. We found that the random positive rate was less than or equal to 6% for any given E11.5 tissue (Extended Data Fig. 9c).

Given the 6% lower bound (random positive rate) and the 10% upper bound (the positive rate in E11.5 limb), we conservatively estimated the genuine positive rate to be 8%. Next, for each E11.5 tissue, we sampled VISTA elements such that the positive rate of the selected VISTA elements in that E11.5 tissue matches the genuine positive rate. Since the positive rate of all VISTA elements in any E11.5 tissue is higher than the estimated genuine positive rate, for a

given E11.5 tissue, we constructed a dataset by randomly selected a subset of positives and all negatives (VISTA elements that are inactive in that tissue) such that its positive rate is equal to 8%, the genuine positive rate. In this way, we are able to get the largest set of VISTA elements while still having positive rate matching genuine positive rate to control for selection bias. To take into account variation introduced by random sampling, we repeated this procedure 100 times for each E11.5 tissue. As a result, for each E11.5 tissue, we generated 100 down-sampled datasets. Using this collection of datasets, we calculated the true positive rate for the top 2500 and top 2501-5000 most confident feDMRs separately (Fig. 4c). Even in these datasets where only 8% of elements are experimentally validated enhancers, the true positive rate of feDMRs remained reasonable: 37%-55% of the VISTA elements that overlapped with top 2500 (3%-7%) most confident feDMRs are experimentally validated enhancers in the matched tissue (Fig. 4c).

To better interpret these results, we included two baseline enhancer prediction method. First, for each tissue, we randomly selected 5,000 genomic bins with matched GC content (sequence content) and PhyloP score (evolution conservation) to the top 5,000 most confident feDMRs. We then calculated the positive rate of the VISTA elements that overlapped with any of these selected regions. We repeated this process 10 times for each E11.5 tissue. The median true positive rate of this baseline method was about 10% (Fig. 4c). We also included a second baseline method. We calculated the positive rate of VISTA elements that did not overlap with any feDMRs or H3K27ac peaks. The positive rate of these VISTA elements was 4-11% depending on tissue examined. The true positive rate of both baseline methods is worse than that of feDMRs.

In summary, we demonstrate that the potential selection bias in the VISTA dataset can be computationally reduced. After controlling for such bias, the feDMRs still achieved a reasonable true positive rate of 37%-55%, more than 5 fold higher than the random positive rate. Therefore, our feDMR annotation should provide a useful resource for future studies of transcriptional regulation in embryonic mouse tissues.

References

7. Lister, R. *et al.* Human DNA methylomes at base resolution show widespread epigenomic differences. *Nature* **462**, 315–322 (2009).

8. Schultz, M. D. *et al.* Human body epigenome maps reveal noncanonical DNA methylation variation. *Nature* **523**, 212–6 (2015).
10. Ziller, M. J. *et al.* Charting a dynamic DNA methylation landscape of the human genome. *Nature* **500**, 477–81 (2013).
17. Luo, C. *et al.* Single-cell methylomes identify neuronal subtypes and regulatory elements in mammalian cortex. **604**, 600–604 (2017).
25. Visel, A., Minovitsky, S., Dubchak, I. & Pennacchio, L. a. VISTA Enhancer Browser - A database of tissue-specific human enhancers. *Nucleic Acids Res.* **35**, 88–92 (2007).
30. Mo, A. *et al.* Epigenomic Signatures of Neuronal Diversity in the Mammalian Brain. *Neuron* **86**, 1369–1384 (2015).
68. Calo, E. & Wysocka, J. Modification of enhancer chromatin: what, how and why? *Mol. Cell* **49**, 10.1016/j.molcel.2013.01.038 (2013).
79. Schroeder, D. I., Lott, P., Korf, I. & LaSalle, J. M. Large-scale methylation domains mark a functional subset of neuronally expressed genes. *Genome Res.* **21**, 1583–1591 (2011).
80. Schroeder, D. I. *et al.* The human placenta methylome. *Proc. Natl. Acad. Sci. U. S. A.* **110**, 6037–6042 (2013).
81. Berman, B. P. *et al.* Regions of focal DNA hypermethylation and long-range hypomethylation in colorectal cancer coincide with nuclear lamina-associated domains. *Nat. Genet.* **44**, 40–46 (2011).
82. Hon, G. C. *et al.* Global DNA hypomethylation coupled to repressive chromatin domain formation and gene silencing in breast cancer. *Genome Res.* **22**, 246–258 (2012).
83. Cannon, M. V, Pilarowski, G., Liu, X. & Serre, D. Extensive Epigenetic Changes Accompany Terminal Differentiation of Mouse Hepatocytes After Birth. *G3 Genes, Genomes, Genet.* (2016). doi:10.1534/g3.116.034785
84. Guo, Y. *et al.* Relationships between Hematopoiesis and Hepatogenesis in the Midtrimester Fetal Liver Characterized by Dynamic Transcriptomic and Proteomic Profiles. *PLoS One* **4**, e7641 (2009).
85. Medvinsky, A., Rybtsov, S. & Taoudi, S. Embryonic origin of the adult hematopoietic system: advances and questions. *Development* **138**, 1017–1031 (2011).
86. Magnani, L., Eeckhoute, J. & Lupien, M. Pioneer factors: directing transcriptional regulators within the chromatin environment. *Trends Genet.* **27**, 465–474 (2011).
87. Sherwood, R. I. *et al.* Discovery of directional and nondirectional pioneer transcription factors by modeling DNase profile magnitude and shape. *Nat Biotech* **32**, 171–178 (2014).
88. Nepveu, A. Role of the multifunctional CDP/Cut/Cux homeodomain transcription factor in regulating differentiation, cell growth and development. *Gene* **270**, 1–15 (2001).
89. Chuong, E. B., Elde, N. C. & Feschotte, C. Regulatory activities of transposable elements: from conflicts to benefits. *Nat Rev Genet* **18**, 71–86 (2017).
90. Vierstra, J. *et al.* Mouse regulatory DNA landscapes reveal global principles of cis-regulatory evolution. **346**, 1007–1013 (2014).
