# Supplementary material for: Spatiotemporal DNA methylome dynamics of the developing mouse fetus
Source: Nature. 2020 Jul 29;583(7818):752–9. doi: 10.1038/s41586-020-2119-x (PMC7398276; doi:10.1038/s41586-020-2119-x)
Supplement: Supplementary file 2 — Reporting Summary [file 41586_2020_2119_MOESM2_ESM.pdf]

nature research

Corresponding author(s): Joseph R. Ecker

Last updated by author(s): Mar 7, 2019

Reporting Summary

Nature Research wishes to improve the reproducibility of the work that we publish. This form provides structure for consistency and transparency in reporting. For further information on Nature Research policies, see Authors & Referees and the Editorial Policy Checklist.

Statistics

For all statistical analyses, confirm that the following items are present in the figure legend, table legend, main text, or Methods section.

n/a	Confirmed	
☐	☒	The exact sample size (n) for each experimental group/condition, given as a discrete number and unit of measurement
☐	☒	A statement on whether measurements were taken from distinct samples or whether the same sample was measured repeatedly
☐	☒	The statistical test(s) used AND whether they are one- or two-sided<br>*Only common tests should be described solely by name; describe more complex techniques in the Methods section.*
☐	☒	A description of all covariates tested
☐	☒	A description of any assumptions or corrections, such as tests of normality and adjustment for multiple comparisons
☐	☒	A full description of the statistical parameters including central tendency (e.g. means) or other basic estimates (e.g. regression coefficient) AND variation (e.g. standard deviation) or associated estimates of uncertainty (e.g. confidence intervals)
☐	☒	For null hypothesis testing, the test statistic (e.g. F, t, r) with confidence intervals, effect sizes, degrees of freedom and P value noted<br>*Give P values as exact values whenever suitable.*
☒	☐	For Bayesian analysis, information on the choice of priors and Markov chain Monte Carlo settings
☒	☐	For hierarchical and complex designs, identification of the appropriate level for tests and full reporting of outcomes
☐	☒	Estimates of effect sizes (e.g. Cohen's d, Pearson's r), indicating how they were calculated

Our web collection on statistics for biologists contains articles on many of the points above.

Software and code

Policy information about availability of computer code

Data collection	No software was used for data collection.
Data analysis	methylpy (1.0.2) was used for processing WGBS data. It is available at https://github.com/yupenghe/methylpy. REPTILE (1.0) algorithm was used to predict fetal enhancer (linked DMRs) and it is available at https://github.com/yupenghe/REPTILE. The custom code were written mainly for drawing figures and are available at https://github.com/yupenghe/encode_dna_dynamics. Deeptools2 (2.3.1) and R (3.3.1) was also used for drawing figures. Other tools include MACS (1.4.2), and MACS2 (2.1.1.20160309) for peak calling, RSEM (1.2.23) for quantifying gene expression, bowtie (1.1.2), bwa (0.7.10) and STAR (2.4.0k) for mapping , picard tools (1.9.2) for removing PCR duplicates, and bedtools (2.27.1) for processing BED files.

For manuscripts utilizing custom algorithms or software that are central to the research but not yet described in published literature, software must be made available to editors/reviewers. We strongly encourage code deposition in a community repository (e.g. GitHub). See the Nature Research guidelines for submitting code & software for further information.

Data

Policy information about availability of data

All manuscripts must include a data availability statement. This statement should provide the following information, where applicable:

- Accession codes, unique identifiers, or web links for publicly available datasets
- A list of figures that have associated raw data
- A description of any restrictions on data availability

The data that support these findings are publicly accessible at https://www.encodeproject.org/ and http://neomorph.salk.edu/ENCODE_mouse_fetal_development.html. Additional RNA-seq datasets for forebrain, midbrain, hindbrain and liver are available at the NCBI Gene Expression Omnibus (GEO) (accession GSE100685). ATAC-seq data for mouse embryonic stem cells is available at GEO (accession GSE113592). Further details describing the data used in this study can be found in Supplemental Tables 1 and 2.

Field-specific reporting

Please select the one below that is the best fit for your research. If you are not sure, read the appropriate sections before making your selection.

☒ Life sciences ☐ Behavioural & social sciences ☐ Ecological, evolutionary & environmental sciences

For a reference copy of the document with all sections, see nature.com/documents/nr-reporting-summary-flat.pdf

Life sciences study design

All studies must disclose on these points even when the disclosure is negative.

Sample size	Each experiment has two biological replicates. The tissue material used for each experiment was from pooled samples from 15-120 embryos or P0 pubs or adult mice. The number of embryos or P0 pups or adult mice collected was determined by whether the materials were sufficient for genomic assays.
Data exclusions	No data was excluded except for samples that failed ENCODE WGBS QC: https://www.encodeproject.org/wgbs/
Replication	Replication Each experiment has two biological replicates and the findings are reproducible.
Randomization	No randomization. Randomization was not feasible given the scale of this study.
Blinding	No blinding, Blinding was not feasible given the scale of this study.

Reporting for specific materials, systems and methods

We require information from authors about some types of materials, experimental systems and methods used in many studies. Here, indicate whether each material, system or method listed is relevant to your study. If you are not sure if a list item applies to your research, read the appropriate section before selecting a response.

Materials & experimental systems

n/a	Involved in the study	
☒	☐	Antibodies
☒	☐	Eukaryotic cell lines
☒	☐	Palaeontology
☐	☒	Animals and other organisms
☒	☐	Human research participants
☒	☐	Clinical data

Methods

n/a	Involved in the study	
☒	☐	ChIP-seq
☒	☐	Flow cytometry
☒	☐	MRI-based neuroimaging

Animals and other organisms

Policy information about studies involving animals; ARRIVE guidelines recommended for reporting animal research

Laboratory animals	Mouse fetal tissues were dissected from embryos of different developmental stages from female C57Bl/6N Mus musculus animals. Animals, used for obtaining tissue materials from E14.5 and P0 stages, were purchased from both Charles River Laboratories (C57BL/6NCrl strain) and Taconic Biosciences (C57BL/6NTac strain). For tissues of remaining developmental stages, animals (C57BL/6NCrl strain) were purchased from Charles River Laboratories.
Wild animals	This study does not involve wild animals
Field-collected samples	This study does not involve samples collected from field
Ethics oversight	All animal work was reviewed and approved by the Lawrence Berkeley National Laboratory Animal Welfare and Research Committee or the University of California, Davis Institutional Animal Care and Use Committee.

Note that full information on the approval of the study protocol must also be provided in the manuscript.
