# Supplementary material for: Spatiotemporal DNA methylome dynamics of the developing mouse fetus
Source: Nature. 2020 Jul 29;583(7818):752–9. doi: 10.1038/s41586-020-2119-x (PMC7398276; doi:10.1038/s41586-020-2119-x)
Supplement: Supplementary file 3 — This file contains the detailed tissue excision procedure for all embryonic and postnatal mouse tissues surveyed in this study. It includes the protocols of experiment preparation, embryo collection, embryo stage assessing and tissue excision/preparation. This document also includes the specific anatomical definition of tissues on every developmental stage. [file 41586_2020_2119_MOESM3_ESM.pdf]

General Tissue Excision Procedures for Embryonic/Postnatal Mouse Tissues

Preparations

Dry ice ethanol bath is set up to flash-freeze excised tissues; ice bucket is prepared to keep the embryos cold while tissues are being collected.

Culling: For embryonic mice, only one pregnant female at a time is euthanized by CO2 asphyxiation, and undergoes terminal dissection for removing embryos.
For postnatal mice (P0), one litter of newborn pups is euthanized at a time with an overdose of isoflurane in a fume hood, followed by decapitation and terminal dissection for tissue collection. While the tissues from an embryo or pup are being collected, the rest of the embryos or euthanized pups of the litter are kept on ice.

Collecting embryos

Euthanize a pregnant female mouse. Grab skin below the center of the belly with forceps and cut through the skin only, not the underlying abdominal cavity. Cut along the ventral midline of the mouse stopping when even with the shoulders. Gently pull at the skin to separate it from the underlying abdominal and thoracic cavities. Cut into the abdominal cavity following the same line you cut along through the skin. Remove the sac containing the string of embryos and remove the embryos by gently cutting away the outer membrane. Perform staging, followed by tissue collection from embryos that meet staging criteria.

Assessing Stage

All staging procedures follow standard procedures/definitions/landmarks as described in "The House Mouse, Atlas of Embryonic Development" by K. Theiler (Springer, 1987, http://www.emouseatlas.org/emap/ema/theiler_stages/house_mouse/book.html).
Generally, for embryonic stages e11.5 and later, only embryos that are within +/- 0.5 days of embryonic development (as assessed by developmental landmarks) are used. Embryos that fall outside this stage window are discarded. Detailed descriptions of all stages can be found in Theiler (1987). Briefly, main features that will be assessed are:

E10.5: Deep lens indentation is one of the distinguishing features of this stage. The 1st branchial arch is conspicuously divided into maxillary and mandibular components. Limb buds are becoming increasingly prominent; forelimb more so than the hind limb bud. See http://www.emouseatlas.org/emap/ema/theiler_stages/StageDefinition/Theiler/ts17%20%20from%20Theiler.pdf for details.

E11.5: Limb buds are round, but no digit precursor structures visible. The forelimb is divided into two regions; the proximal part which consists of future limb-girdle and arm, and the peripheral part which forms a circular or paddle-shaped hand plate (anterior footplate). The posterior limb buds are not yet divided into leg and foot. See http://www.emouseatlas.org/emap/ema/theiler_stages/StageDefinition/Theiler/ts19%20%20from%20Theiler.pdf for details.

E12.5: A distinct characteristic of this stage relates to the appearance of the hand and foot plates. The hand plates are no longer rounded as observed at e11.5, but have developed angles that correspond to future digits, and these are separated by indented 'rays' that correspond to the digital interzones. The foot plates at e12.5 are only slightly less differentiated than the hand plates. See http://www.emouseatlas.org/emap/ema/theiler_stages/StageDefinition/Theiler/ts20%20%20from%20Theiler.pdf for details

E13.5: The distal borders of the anterior and posterior foot plates are now indented. The degree of indentation is slightly more marked in the hand plate than in the foot plate. In the forelimb, the wrist and elbow are identifiable whereas in the hind limb only the ankle region is distinguishable. The pinna is well formed and directed anteriorly. Five rows of vibrissae (whiskers) are visible. See http://www.emouseatlas.org/emap/ema/theiler_stages/StageDefinition/Theiler/ts21%20%20from%20Theiler.pdf for details.

E14.5: A distinct characteristic of this stage is the separation of individual fingers in the anterior footplate. The posterior footplate shows deep indentations between developing toes but separation is not yet observable. See http://www.emouseatlas.org/emap/ema/theiler_stages/StageDefinition/Theiler/ts22%20%20from%20Theiler.pdf for details.

E15.5: Inter-digital indentation in the hand plate and the foot plate is complete and the webbing disappears making the individual digits more clearly defined. The bending of the knee and elbow is apparent. Hair follicles are present in the cephalic region but not at the periphery of the vibrissae. There is a marked difference in the shape of the back (post-cranial vertebral axis) which is no longer curved and the anterior part of the back is straight. See http://www.emouseatlas.org/emap/ema/theiler_stages/StageDefinition/Theiler/ts24%20

%20from%20Theiler.pdf for details.

E16.5: The external distinguishing features include considerably more prominent pads on the palmar and plantar surfaces of forelimbs and hindlimbs respectively, compared to earlier embryonic stages. Furthermore, the hindlimbs of e16.5 embryos have a pronounced bend (articulation) at the ankles, forming the heel. The fingers and toes are parallel. The skin in the neck, trunk and limbs has thickened and formed large number of wrinkles. The eyelids have fused and appear closed. The pinna almost completely covers the external auditory meatus (ear canal). For additional details see http://www.emouseatlas.org/emap/ema/theiler_stages/StageDefinition/Theiler/ts25%20%20from%20Theiler.pdf for details.

For postnatal stages, time from birth is determined. Postnatal day 0 (P0) is defined as the first day newborn mice are found (checking cages in 24hr intervals, so mice are between 0 and 24 hrs from birth).

General Tissue Excision/Preparation Procedures

- A single embryo or euthanized pup is placed in a Petri dish in ice-cold PBS under an SMZ stereomicroscope.
- The body cavity is opened up to collect tissues using microdissection forceps, blades or microscissors as needed.
- From e10.5 embryos the following 6 tissues are collected: forebrain, midbrain, hindbrain, heart, limbs, and craniofacial tissue (see below for additional definitions/comments).
- From e11.5 embryos, the following 8 tissues are collected: forebrain, midbrain, hindbrain, heart, liver, limbs, craniofacial tissue and neural tube (see below for additional definitions/comments).
- From e12.5 embryos, the following 8 tissues are collected: forebrain, midbrain, hindbrain, heart, liver, limbs, craniofacial tissue and neural tube (see below for additional definitions/comments).
- From e13.5 embryos, the following 8 tissues are collected: forebrain, midbrain, hindbrain, heart, liver, limbs, craniofacial tissue and neural tube (see below for additional definitions/comments).
- From e14.5 embryos, the following 12 tissues are collected: forebrain, midbrain, hindbrain, heart, lung, liver, intestine, stomach, kidney, limbs, craniofacial tissue and neural tube (see below for additional definitions/comments).
- From e15.5 embryos, the following 12 tissues are collected: forebrain, midbrain, hindbrain, heart, lung, liver, intestine, stomach, kidney, limbs, craniofacial tissue and neural tube (see below for additional definitions/comments).
- From e16.5 embryos, the following 9 tissues are collected: forebrain, midbrain,

hindbrain, heart, lung, liver, intestine, stomach, and kidney. (see below for additional definitions/comments).
- For P0 pups, the following 9 tissues are collected: forebrain, midbrain, hindbrain, heart, lung, liver, intestine, stomach and kidney (see below for additional definitions/comments).
- For adults (8-9 week old), the following tissues were collected: Cortex, cerebellum, olfactory bulbs, heart, lung, liver, spleen, small intestine, large intestine, kidney, thymus and bone marrow. (see below for additional definitions/comments).

Effort is made to perform the dissections in the shortest possible time and depend on the numbers of embryos or pups per litter, the numbers of tissues being collected or the stages of collection. In all cases the target is to flash-freeze tissue within 1 hour from euthanasia of female/pups (with embryos/pups being kept on ice) and within 15 minutes from start of dissection of each single embryo/pup. Each tissue is flash-frozen as soon as it is excised by dropping it into a pre-labeled tube set up in a dry ice/ethanol bath.

Once all the tissues are collected, and flash frozen they are transferred to a box and stored in -80C freezer until ready to ship.

For the next set of collections, the same procedure is repeated and the excised tissues are added to the tissues collected previously until a pool of tissue samples from 25-35 embryos per tube has been collected.

Pools of samples are shipped on dry ice for further processing (ChIP-seq/RNA-seq etc.) using the World Courier shipping service.

Comments for dissection of specific structures

Generally, tissue descriptions and boundaries of regions (in case of brain) follow standard nomenclature as shown in Theiler (1987). Specific comments/clarifications include:

E10.5

Forebrain – this includes the telencephalon (cortex and basal ganglia) and the diencephalon (hypothalamus and thalamus)
Midbrain – as "mesencephalon in Theiler (1987)
Hindbrain – as "rhombencephalon" in Theiler (1987)
Limb- this includes pooled fore- and hindlimb buds.
Craniofacial – this includes the entire craniofacial region including frontonasal and

lateral nasal processes, maxillary and mandibular components of the first branchial arch, second branchial arch, general facial mesenchyme. It does not include the eyes.
Heart – this includes the entire heart, i.e. all four chambers.

E11.5

Forebrain – this includes the telencephalon (cortex and basal ganglia) and the diencephalon (hypothalamus and thalamus)
Midbrain – as “mesencephalon in Theiler (1987)
Hindbrain – as “rhombencephalon” in Theiler (1987)
Neural tube – this includes the neural tube from the neural tube-hindbrain boundary to approximately the hindlimb (not the section of the neural tube posterior to the hindlimbs)
Limb- this includes the distal (autopod) and proximal (stylopod/zeugopod) regions of the limbs. Fore- and hindlimbs are pooled.
Craniofacial – this includes the entire craniofacial region including frontonasal and lateral nasal processes, maxillary and mandibular components of the first branchial arch, second branchial arch, general facial mesenchyme. It does not include the eyes.
Heart – this includes the entire heart, i.e. all four chambers.
Liver – all lobes, as in Theiler (1987)

E12.5

Forebrain – this includes the telencephalon (cortex and basal ganglia) and the diencephalon (hypothalamus and thalamus)
Midbrain – as “mesencephalon in Theiler (1987)
Hindbrain – as “rhombencephalon” in Theiler (1987)
Neural tube – this includes the neural tube from the neural tube-hindbrain boundary to approximately the hindlimb (not the section of the neural tube posterior to the hindlimbs)
Limb- this includes the distal (autopod) and proximal (stylopod/zeugopod) regions of the limbs. Fore- and hindlimbs are pooled.
Craniofacial – this includes the entire craniofacial region including frontonasal and lateral nasal processes, maxillary and mandibular components of the first branchial arch, second branchial arch, general facial mesenchyme. It does not include the eyes.
Heart – this includes the entire heart, i.e. all four chambers.
Liver – all lobes, as in Theiler (1987)

E13.5

Forebrain – this includes the telencephalon (cortex and basal ganglia) and the diencephalon (hypothalamus and thalamus)
Midbrain – as “mesencephalon in Theiler (1987)
Hindbrain – as “rhombencephalon” in Theiler (1987)

Neural tube – this includes the neural tube from the neural tube-hindbrain boundary to approximately the hindlimb (not the section of the neural tube posterior to the hindlimbs)
Limb – this includes the distal (autopod) and proximal (stylopod/zeugopod) regions of the limbs. Fore- and hindlimbs are pooled.
Craniofacial – this includes the entire craniofacial region including frontonasal and lateral nasal processes, maxillary and mandibular components of the first branchial arch, second branchial arch, general facial mesenchyme. It does not include the eyes.
Heart – this includes the entire heart, i.e. all four chambers.
Liver – all lobes, as in Theiler (1987)

E14.5

Forebrain – this includes the telencephalon (cortex and basal ganglia) and the diencephalon (hypothalamus and thalamus)
Midbrain – as in Theiler (1987) Not specifically labeled as such in reference, but same boundaries as e11.5
Hindbrain – as in Theiler (1987) Not specifically labeled as such in reference, but same boundaries as e11.5
Neural tube – this includes the neural tube from the neural tube-hindbrain boundary to approximately the hindlimb (not the section of the neural tube posterior to the hindlimbs)
Limb- this includes the distal (autopod) and proximal (stylopod/zeugopod) regions of the limbs. Fore- and hindlimbs are pooled.
Craniofacial – this includes the entire craniofacial region including the derivatives of the frontonasal and lateral nasal processes, maxillary and mandibular components, general facial mesenchyme. It does not include the eyes.
Heart – this includes the entire heart, i.e. all four chambers.
Liver – all lobes, as in Theiler (1987)
Intestine – this includes all sections of the small and large intestines
Kidney – this includes the entire kidney, but not the adjacent adrenal gland
Lung – as in Theiler (1987) - only small subregion shown in reference, overall obvious from tissue appearance and location
Stomach – as in Theiler (1987) same position as indicated in reference for e13.5, Fig. 208

E15.5

Forebrain – this includes the telencephalon (cortex and basal ganglia) and the diencephalon (hypothalamus and thalamus)
Midbrain – as in Theiler (1987) Not specifically labeled as such in reference, but same boundaries as e11.5
Hindbrain – as in Theiler (1987) Not specifically labeled as such in reference, but same boundaries as e11.5
Neural tube – this includes the neural tube from the neural tube-hindbrain boundary to approximately the hindlimb (not the section of the neural tube posterior to the hindlimbs)

Limb – this includes the distal (autopod) and proximal (stylopod/zeugopod) regions of the limbs. Fore- and hindlimbs are pooled.
Craniofacial – this includes the entire craniofacial region including the derivatives of the frontonasal and lateral nasal processes, maxillary and mandibular components, general facial mesenchyme. It does not include the eyes.
Heart – this includes the entire heart, i.e. all four chambers.
Liver – all lobes, as in Theiler (1987) see Fig 252 in reference
Intestine – this includes all sections of the small and large intestines
Kidney – this includes the entire kidney, but not the adjacent adrenal gland
Lung – as in Theiler (1987) only small subregion shown in reference, overall obvious from tissue appearance and location
Stomach – as in Theiler (1987) labeled Ma in Fig. 247 of reference

E16.5

Forebrain – this includes the telencephalon (cortex and basal ganglia) and the diencephalon (hypothalamus and thalamus)
Midbrain – as in Theiler (1987) Not specifically labeled as such in reference, but same boundaries as e11.5
Hindbrain – as in Theiler (1987) Not specifically labeled as such in reference, but same boundaries as e11.5
Heart – this includes the entire heart, i.e. all four chambers.
Liver – all lobes, as in Theiler (1987) see Fig 252 in reference
Intestine – this includes all sections of the small and large intestines
Kidney – this includes the entire kidney, but not the adjacent adrenal gland
Lung – as in Theiler (1987) only small subregion shown in reference, overall obvious from tissue appearance and location
Stomach – as in Theiler (1987) labeled Ma in Fig. 245 of reference

P0

Forebrain – this includes the telencephalon (cortex and basal ganglia) and the diencephalon (hypothalamus and thalamus)
Midbrain - as described for E17.5 in Theiler (1987)
Hindbrain - Cerebellum plus brain stem (the latter not specifically labeled in reference) Theiler (1987)
Heart – this includes the entire heart, i.e. all four chambers.
Liver – all lobes, as described for E17.5 in Theiler (1987)
Intestine – this includes all sections of the small and large intestines. Intestines are flushed with PBS buffer to remove all contaminating milk-/food-derived contents. Intestines are visually inspected to be free of remaining contaminants.
Kidney – this includes the entire kidney, but not the adjacent adrenal gland
Lung - as described for E17.5 in Theiler (1987)
Stomach – from esophageal sphincter to pyloric sphincter

Adult

Cerebral Cortex - this includes both the cortical halves of the cerebrum
Cerebellum - this includes the entire cerebellum
Olfactory bulbs - this includes both olfactory lobes
Thymus - this includes both thymus lobes
Heart - this includes all four chambers of the heart.
Liver - this includes all lobes of the liver
Lungs - this includes all parts of the lung
Small Intestine - this includes all sections of the small intestine. Small intestines are flushed with PBS buffer to remove all contaminating food-derived contents. Small intestines are visually inspected to be free of remaining contaminants.
Large intestine - This includes all sections of large intestine except the cecum. Large intestines are flushed with PBS buffer to remove all contents and are visually inspected to be free of remaining contaminants
Kidney - this includes both the kidneys, but not the adjacent adrenal gland
Spleen - this includes the entire spleen
Bone Marrow - Bone marrow was extracted from the mouse femur and the tibia using the referenced protocol (Amend, S. R., Valkenburg, K. C., Pienta, K. J. Murine Hind Limb Long Bone Dissection and Bone Marrow Isolation. J. Vis. Exp. (110), e53936, doi:10.3791/53936 (2016).
