# Supplementary material for: Spatiotemporal DNA methylome dynamics of the developing mouse fetus
Source: Nature. 2020 Jul 29;583(7818):752–9. doi: 10.1038/s41586-020-2119-x (PMC7398276; doi:10.1038/s41586-020-2119-x)
Supplement: Supplementary file 4 — This file describes the detailed protocols of tissue fixation, sonication and DNA extraction, including several steps: pulverization of tissue, cross-linking of tissues, sonication of tissues and DNA isolation (precipitation and chromatin fragmentation check). [file 41586_2020_2119_MOESM4_ESM.pdf]

Ren Lab ENCODE Tissue Fixation and Sonication Protocol

Pulverization of Tissue

Note: Ensure the samples are kept frozen on dry ice throughout pulverization.

1. Pour liquid nitrogen into a mortar and pestle.
2. Remove tissue from tube and place into liquid nitrogen with a clean cold spatula.
3. Grind up the sample with liquid nitrogen using the mortar and pestle.
4. Use a clean cold spatula to scoop ground tissue back to the original tube.
5. Place the sample back onto the dry ice.
6. Clean the mortar and pestle with 10% bleach and 70% ethanol before using it for the next sample.

Cross-linking of Tissue

7. Transfer tissue into a 15 mL conical tube using a clean spatula or pipette tip.
8. Add cold 1x PBS to 5 mL.
9. Add 0.5 mL crosslinking buffer (see recipe below) and rotate the tube at room temperature for 20 min.

Reagent	Stock Concentration	Final Concentration	Volume for 5 mL
NaCl	5 M	0.1 M	0.1 mL
EDTA	0.5 M	1 mM	10 µL
EGTA	0.5 M	0.5 mM	5 µL
Hepes pH8.0	1 M	50 mM	0.25 mL
Formaldehyde	37%	11%	1.5 mL
dH2O	--	--	3.14 mL

10. Stop the crosslinking reaction by adding 0.275 mL 2.5 M glycine to a final concentration of 0.125 M.
11. Rotate at room temperature for 5 min.
12. Centrifuge samples at low speed (15min at 2000 x g).
13. Decant the supernatant and wash once with cold 1X PBS.
14. Centrifuge at low speed (10 min at 2500 x g).
15. Decant the supernatant.
16. Store cells at -80°C or proceed to sonication.

Sonication of Tissue

17. Resuspend fixed cells in 50 µL lysis buffer (see recipe below) and incubate for 10 min on ice

Reagent	Stock Concentration	Final Concentration	Volume for 500 µL
SDS	10%	1%	50 µL
Tris-HCl, pH 8.0	1 M	50 mM	25 µL
EDTA	0.5 M	20 mM	20 µL
cOmplete EDTA-free protease inhibitor (Roche, Cat#05056489001)	50x	1x	10 µL
dH2O	--	--	395 µL

18. Dilute to 500 µL using cold 1x TE.
19. Using a Branson Sonifier 450, sonicate each sample for 15-50 cycles (15 sec ON, 45 sec OFF at power 3). Number of cycles depends on the type of tissue and the size of the pellet.
20. Pellet debris at 2000 x g for 15 min in 4°C.
21. Transfer supernatant to a new tube.
22. Measure DNA concentration (e.g. using NanoDrop).
23. Remove 20 µL chromatin for fragmentation check (below). The remaining chromatin can be used immediately for immunoprecitpation (see “Chromatin Immunoprecipitation Protocol”) or flash frozen in dry ice or liquid nitrogen and stored at -80°C.

DNA Isolation – Precipitation and Chromatin Fragmentation Check

24. Add 20 µL chromatin to 130 µL ChIP elution buffer (see recipe below), and incubate overnight at 65°C to reverse crosslinks.
25. Add 250 µL 1x TE.
26. Add 8 µL of 10 mg/mL RNase A (final conc. = 0.2 mg/mL), and incubate at 37°C for 1 hr.
27. Add 8 µL of 20 mg/mL Proteinase K (final conc. = 0.4 mg/mL), and incubate at 55°C for 1 hr.
28. Prepare one Phase Lock tube (5 Prime, Cat#2302820) per IP by spinning down the gel to the bottom of the tube at 20,000 x g for 1 min.
29. Add 400 µL Phenol: Chloroform: Isoamyl Alcohol (25:24:1) alcohol to each Phase Lock tube.
30. Add sample to Phase Lock tube and invert the tube until the contents turn white.
31. Spin for 4 min at max speed. Note: if aqueous phase is cloudy, extract again.
32. Transfer aqueous layer to a new 1.7 mL Eppendorf tube.
33. Add 16 µL of 5 M NaCl (final conc. = 200 mM) and 2 µL of 20 mg/mL glycogen (40 µg total) to each sample and vortex or pipet up and down to mix.
34. Add 920 µL cold 100% EtOH and vortex briefly.
35. Incubate at -80°C for 30 min or until frozen solid.
36. Spin at 20,000 x g for 15 min at 4°C.
37. Wash pellet with 1 mL cold 70% EtOH and spin for 5min at 4°C at 20,000 x g.
38. Remove EtOH using a pipet without disturbing the DNA pellet.
39. Dry the pellet for 5 min at room temperature.
40. Thoroughly resuspend the pellet in 50 µL 10 mM Tris.
41. Measure DNA concentration (e.g. using NanoDrop) and check the size of the fragmentation on an agarose gel. Average size of chromatin fragmentation should be 200bp.
42. Store the remaining material at -20°C until needed. It can be prepared for sequencing and used as an input-control.

**Reagent**	**Stock Concentration**	**Final Concentration**	**Volume for 50 mL**
Tris, pH 8.0	1 M	10mM	0.5 mL
EDTA	0.5 M	1mM	0.1 mL
SDS	10%	1%	5 mL
dH2O	--	--	44.4 mL
